# Implementation of a Mobile Digital Tool Supporting Medication for Opioid Use Disorder Treatment Improves Retention: Stepped-Wedge Cluster Randomized Controlled Trial

**DOI:** 10.2196/83346

**Published:** 2025-12-22

**Authors:** Jorge E Palacios, Robert Sherrick, Tim Janssen, Elana Deuble, Sara Lorenzen, Mark Schaefer, Jenna Tregarthen

**Affiliations:** 1 Bright Therapeutics San Francisco, CA United States; 2 Community Medical Services Scottsdale, AZ United States; 3 Department of Behavioral and Social Sciences Brown University School of Public Health Providence, RI United States

**Keywords:** digital intervention, opioid use disorder, medication for opioid use disorder, treatment retention, stepped-wedge cluster randomized trial, blended treatment

## Abstract

**Background:**

Despite its proven efficacy, retention in medication for opioid use disorder (MOUD) remains low, with structural and systemic barriers—such as access to care and treatment setting—alongside individual factors, including personalization and motivation, contributing to high rates of discontinuation. Digital interventions offer a promising approach to address many of these barriers; however, robust evidence for their effectiveness in improving retention and engagement with treatment remains scarce.

**Objective:**

This study aims to evaluate the impact of Recovery Connect—a white-labeled version of Recovery Path and a digital remote patient monitoring app used as part of a blended treatment model for opioid use disorder—on patient retention, treatment continuance, and medication adherence.

**Methods:**

A stepped-wedge cluster randomized trial was conducted across 9 outpatient MOUD clinics, organized into 8 clusters. Clusters were sequentially transitioned from usual care to a digitally enhanced model incorporating Recovery Connect, which provided real-time monitoring, psychoeducational and skill-based content, and messaging between patients and clinicians. The primary outcome was 30-day retention in treatment following exposure (implementation of the app in the clinic), linkage (downloading and connecting to the app), or engagement (levels of app usage). Secondary outcomes included treatment continuance—defined as receiving at least 75% of expected doses—and the number of daily doses taken within the first 3, 7, and 30 days after admission. Cluster-controlled discrete-time survival analyses were conducted, adjusting for patient- and clinic-level covariates.

**Results:**

Patients admitted to clinics that had implemented the app (n=1205) showed increased retention (922/1205, 75.5%) compared with those in clinics that had not (203/319, 63.6%, *P*<.001). Patients who downloaded and linked with a mental health professional on Recovery Connect had an 81.3% likelihood of retention, compared with 72.0% (*P*<.001) among those not linked. Linkage also significantly predicted higher treatment continuance and a greater number of daily doses taken during the first 7 and 30 days (*P*<.001). Low, moderate, and high engagement levels were associated with progressively higher 30-day retention compared with no engagement (*P*<.001).

**Conclusions:**

This study provides evidence that implementing Recovery Connect (Recovery Path) significantly enhances patient retention and treatment continuity in outpatient opioid use disorder care. Early linkage and engagement during the first week were strong predictors of positive outcomes, underscoring the value of early, proactive digital support. These findings reinforce the effectiveness of blended digital-clinical models, aligning with broader evidence that integrating remote monitoring enhances continuity of care and supports recovery. Policy implications include the need for reimbursement mechanisms, workflow integration, and ethical, privacy-preserving implementation to enable scalable and equitable adoption of digital tools in substance use treatment.

**Trial Registration:**

ClinicalTrials.gov NCT07140926; https://clinicaltrials.gov/ct2/show/NCT07140926

## Introduction

Opioid use disorder (OUD) remains a significant public health challenge, characterized by high morbidity, mortality, and substantial societal costs [1,2]. Medications for opioid use disorder (MOUD) have been shown to reduce opioid use and mortality by more than 50% while improving social and health outcomes [3-5]. Yet, despite this efficacy, MOUD retention remains low, with structural and systemic barriers, such as access to care, treatment setting, and referral source, compounding individual patient factors (availability of treatment, personalization, and motivation) and contributing to high rates of discontinuation [6-8].

Digital interventions hold promise for overcoming common barriers such as stigma, limited clinic access, and resource constraints [9]. A recent systematic review highlights the potential of digital and remote interventions to augment traditional substance use treatment, reduce the risk of relapse, and offer interactive contact between patients and clinicians [10]. These technologies may not only facilitate improved retention but also foster patient autonomy and continuous engagement with care.

Preliminary findings from a large-scale feasibility study indicate that Recovery Connect (RC), a digital remote patient monitoring app, successfully facilitated patient-clinician interactions, was well accepted, and was associated with improved 30-day retention [11]. However, robust randomized trial evidence evaluating app-based tools in real-world opioid treatment settings remains limited [10]. This study addresses this gap by rigorously testing the effectiveness of RC implementation through a stepped-wedge cluster randomized design.

The main objectives of this study were (1) to assess the effect on retention of implementing RC in outpatient OUD clinics relative to care as usual, (2) to explore the impact on retention of clinician-patient linkage facilitated by the app, and (3) to examine the association between patient engagement levels and treatment retention.

## Methods

### Study Design

**Figure 1:**
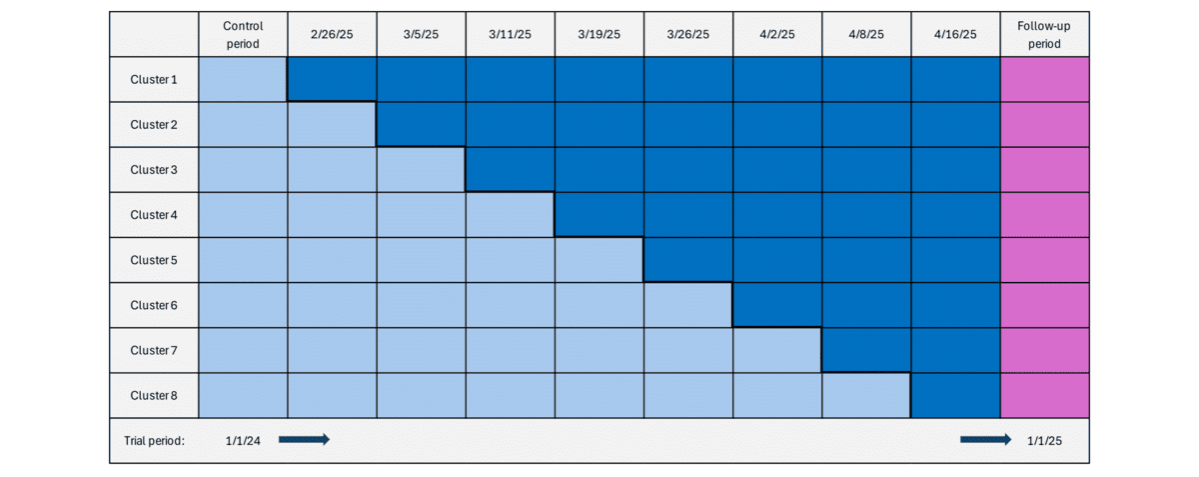
Stepped-wedge crossover design for this study. Colors represent the timing of cluster crossovers—that is, when MHPs received instruction on app use and client linkage. Light blue indicates usual care for MOUD, and dark blue indicates periods after MHP instruction. The follow-up period (purple) extended to 60 days after the final day of recruitment (November 1, 2024) to ensure that the 30-day retention metric could be assessed for all participants (ie, to determine whether any patient initiated a ≥30-day treatment gap within the first 30 days after admission). MHP: mental health professional; MOUD: medication for opioid use disorder.

We conducted a stepped-wedge cluster randomized trial [12] to evaluate the impact of RC on retention and treatment continuance among individuals initiating MOUD. This design allowed for sequential implementation of the intervention, with each cluster transitioning from a control period (usual care), beginning January 1, 2024, to an intervention period (RC), thereby serving as its own control. Clusters were randomized to cross over into the intervention condition at weekly intervals beginning February 26, 2024, through April 16, 2024. We used simple randomization without replacement, generating a random permutation of all clusters in Microsoft Excel (RAND function), sorting clusters by the random number, and assigning them sequentially to the intervention condition. The randomization algorithm and allocation schedule were finalized and archived before enrollment and implementation. To preserve allocation concealment, the random sequence was created by an investigator (RS) not involved in recruitment or delivery, and the crossover dates were released to sites only as required for scheduling. Figure 1 shows the full data collection period alongside the dates for crossover at each clinic. The stepped-wedge approach was selected to facilitate pragmatic implementation, reflecting real-world conditions while preserving the ability to evaluate causal effects. This design also allowed us to account for temporal trends and clinic-level variability through its staggered rollout. As the intervention involved implementing a digital tool within routine care workflows, blinding of care providers and patients was not feasible.

### Ethical Considerations

The trial protocol was approved by the Sterling Institutional Review Board (IRB; ID: 11629). All collected data, including app usage statistics and clinical outcomes, are stored securely in compliance with applicable data protection regulations. Digital data are encrypted and stored on secure servers with restricted access. Clinicians provided informed consent to participate in the study. Patients give consent for their coded data to be used for research purposes as part of their treatment contract, and thus, a waiver of consent was approved by the IRB. All protected health information was removed from study data by the study clinician and co-author (RS) before sharing with the research team for analysis; global identifiers were coded to identify follow-up admissions. No additional costs were incurred by clinicians or patients participating in the study. The implementation of the RC app was part of the rollout at participating clinics and was integrated into existing treatment pathways. No compensation was given to clinicians, as this work fell within their regular duties and the research did not require them to perform any tasks beyond their usual clinical practice. Similarly, patients were not compensated for their involvement in the study through the use of their data.

### Setting and Participants

The study was conducted across 9 outpatient MOUD clinics operated by Community Medical Services (CMS), a network of Opioid Treatment Programs serving urban and rural populations across multiple US states. Participating clinics were located in Montana (Billings, Bozeman, Kalispell, and Missoula), North Dakota (Fargo, Grand Forks, and Minot), and Alaska (Anchorage and Wasilla). Grand Forks and Fargo were grouped into 1 cluster, as Fargo clinicians often treat Grand Forks patients. All clinics provided methadone and buprenorphine treatment under similar protocols, including same-day admissions, observed daily dosing, routine counseling, regular urine drug screening, and medical follow-ups within 7 days of admission and as needed thereafter. Treatment is delivered by mental health professionals (MHPs), whose education spans from high school diplomas to advanced degrees in social work and counseling. Eligible participants were adults (18+) newly admitted to treatment between January 1 and November 1, 2024. Patients were excluded if their dosing suggested a transfer from a non-CMS clinic—namely, if their methadone starting dose exceeded 30 mg before April 1, 2024, or exceeded 50 mg after April 1, 2024—or if they had previously used RC before their most recent admission during the study. For patients with multiple admissions during the study period, their most recent admission was selected. Covariate-adjusted analyses controlled for the presence and number of prior admissions.

Exposure groups were defined by implementation timing and patient app download/linkage to the MHP app, as detailed in the “Intervention” section. To statistically account for patients whose MHP was trained in the use of RC during their ongoing admission, survival analyses used a time-varying indicator of exposure to reflect the day that MHP training in the RC platform occurred; see the “Analysis Plan” section.

### Control Condition: Usual Care

During the control period, all clinics followed standard MOUD protocols. Usual care included in-person dosing as well as take-home dosing per federal guidelines. Counseling was available on-site and included both individual and group therapy sessions. Communication with patients outside the clinic setting was generally limited to phone calls, and no structured digital tools were used to support remote monitoring, messaging, or check-ins. Documentation was completed via electronic health records, but there was no mechanism for providing real-time intervention or behavioral feedback.

### Intervention: Recovery Connect

RC (Version 1.4.3) is a white-labeled version of Recovery Path [13], rebranded to allow partner organizations to maintain brand continuity while leveraging its evidence-based digital infrastructure. Recovery Path (patient version freely available to download on Google Play and the App Store in the United States) is a HIPAA (Health Insurance Portability and Accountability Act)-compliant digital remote patient monitoring platform that supports a blended care model for MOUD by combining in-person treatment with continuous digital engagement. It undergoes continuous, strict quality assurance testing for bugs, performance, and overall user experience, ensuring the ongoing accuracy of clinical content. It enables daily check-ins, access to evidence-based resources and coping strategies, and secure, real-time communication between patients and their care teams. MHPs gain timely insights through real-time data on patient progress, risks, and setbacks, allowing for early intervention and timely containment during periods of heightened distress. Between sessions, MHPs can recommend tailored coping strategies and psychoeducational content, reinforcing accountability and enhancing continuity of care. RC was also integrated into the CMS medical record system to reduce administrative burden on MHPs and improve documentation accuracy through auto-generated case notes.

Implementation involved 2 components: MHP training and patient onboarding. On the day of training assigned for each clinic, all MHPs staffed at that clinic received live virtual training together on app features, linkage procedures, and app-based communication strategies. Materials included step-by-step guides, video tutorials, and ongoing technical support. The training was deliberately designed to accommodate differing levels of prior experience, ensuring that every MHP gained the skills needed to use the app effectively in clinical settings. Training lasted approximately 2 hours, and app rollout began immediately afterward, at which point MHPs—now considered “trained” for study and analysis purposes—were instructed to help newly admitted patients download the RC app and link via a QR code during onboarding. MHPs were also asked to show patients key features of RC, including daily check-ins, homework activities, and the messaging feature.

For primary analyses, we operationalized 3 key constructs:

- Exposure: Patients were considered “exposed” if MHPs at their clinic had completed training in the RC platform before or during the patient’s ongoing admission.
- Linkage: Patients were considered “linked” if they had downloaded the RC app and were connected to an MHP trained in the use of RC at any point during the patient’s admission process.
- Engagement: Defined as meaningful usage (not merely opening the app) during the first week postlinkage. This included messages sent by clinicians and patients, as well as patient self-monitoring entries (a core feature supporting patients in tracking cravings, triggers, motivation, anxiety, depression, sleep patterns, upcoming risky events, interpersonal issues, and relapse prevention planning). The 1-week total of messages sent + messages received + self-monitoring entries completed was calculated. Compared with no engagement, totals below 5 represented minimal engagement, 5-9 represented moderate engagement, and 10 or more represented high engagement.

These categories allowed us to examine both the impact of clinic-level implementation (exposure) and the patient-level uptake and use of the app (linkage and engagement). A full overview of data transformations and underlying statistical considerations is provided in Multimedia Appendix 1, alongside the code syntax for all analyses. Additional details on timing and classification are described in the “Analysis Plan” section.

### Measures

#### Primary Outcome

The primary outcome was treatment retention. In line with federal guidelines, patients who leave care can be readmitted within 30 days without restarting the intake process [14]. Therefore, a patient was considered to have dropped out—and thus not retained—if a 30-day gap in treatment began within the first 30 days after intake. The first day of that gap was considered the discontinuation date.

#### Secondary Outcomes

##### Treatment Continuance

This is a binary measure defined as receiving at least 75% of expected doses within the first 30 days following intake, and at least one dose administered during the final week of that period (ie, days 24-30 postintake).

##### 3-, 7-, and 30-Day Doses

This refers to the number of confirmed daily MOUD doses administered during the first 3, 7, and 30 days after intake, respectively. Dose analyses were limited to patients retained for at least the corresponding number of days. These outcomes provide a more granular view of early medication adherence patterns. Confirmed doses included both in-clinic administrations and verified take-home doses, based on standard documentation protocols across clinics.

### Covariates

All covariates were assessed at the time of patient admission and included age, gender, ethnicity, presence of any fentanyl or amphetamine use, presence of moderate-to-severe depression or anxiety, unemployment or unhoused status, any prior CMS admissions, number of prior CMS admissions, and primary MOUD medication (methadone or buprenorphine).

### Analysis Plan

We used intent-to-treat analyses, in which all patients from the trial dataset whose sites engaged in training activities were included, unless previously excluded based on the study’s exclusion criteria. The effects of exposure and linkage on odds of retention were tested using cluster-controlled discrete-time survival analyses, accounting for the nested nature of patients within sites. In these analyses, the likelihood of successful client retention on a given day—provided loss to follow-up had not occurred earlier—was regressed on prior-day exposure or linkage status. Loss to follow-up was defined as the event (1), retention as the absence of the event (0), and days following loss to follow-up were coded as missing data [15]. The effects of app engagement on retention, as well as the effects of provider intervention exposure and patient linkage on secondary outcomes, were assessed using cluster-controlled logistic and linear regression analyses. Effects of exposure were examined across all patients based on their degree of exposure. Effects of linkage and engagement were examined across all patients, as well as among those fully exposed. This approach allowed us to study both the pragmatic, system-level impact of linkage and engagement under routine conditions—including partial exposure and implementation variability—and to analyze the fully exposed subgroup to isolate effects under conditions of implementation fidelity.

Main effects analyses were tested across a series of models that estimated the effect of condition (both unadjusted and adjusted for covariates), following a moderator-first approach to ensure treatment effects were consistent across strata of predictors [16]. The effects of exposure, linkage, and engagement were first modeled as a function of their main effects, as well as their interaction with each covariate in separate models. We did not hypothesize specific interaction effects; therefore, only interactions that were significant at Benjamini-Hochberg–corrected *P* values were considered nonspurious and were retained in the final covariate-adjusted model that included all covariates [17].

All MHP- and patient-level analyses were conducted in Mplus 8.9 [18] using full-information maximum likelihood estimation with a robust sandwich estimator to allow nonparametric adjustment for clustering by site, employing a design-based adjustment to the estimation of SEs for nesting and for potential violations of multivariate normality [19]. Full-information maximum likelihood allows for estimation under the assumption of missing at random, meaning that valid inferences can be drawn in the presence of missing outcome data if known predictors of missingness are included in the model [20].

## Results

### Overview

**Figure 2:**
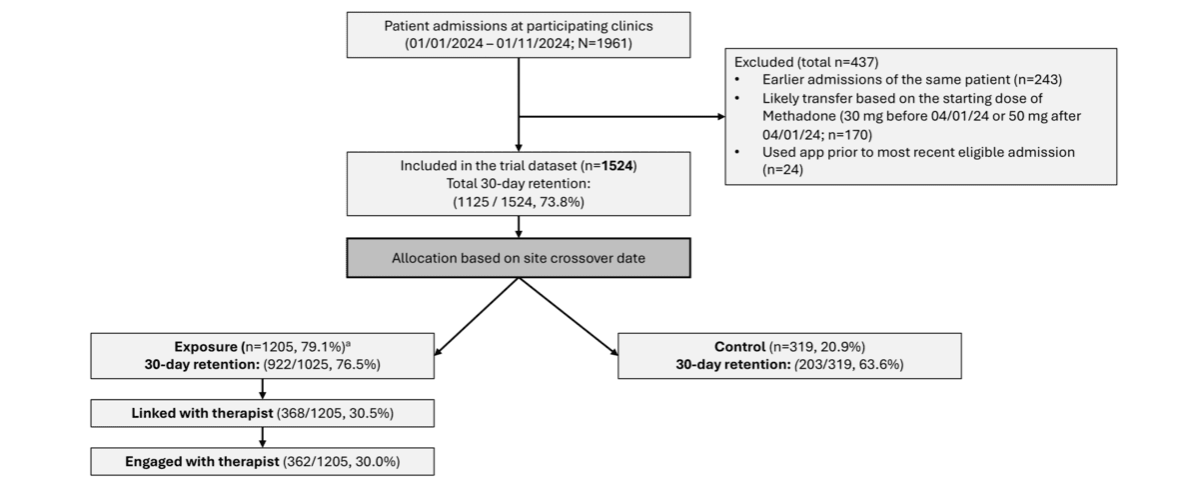
CONSORT (Consolidated Standards of Reporting Trials) flowchart. ª Patients whose clinic crossed over while they remained in treatment were classified under the exposure allocation.

A CONSORT (Consolidated Standards of Reporting Trials) diagram (Figure 2; see Multimedia Appendix 2 for the checklist) illustrates participant flow, including exclusions, exposure classification, linkage status, and retention of eligible patients by condition. A total of 1524 patients were allocated to study arms. Sociodemographic characteristics are summarized in Table 1. Among exposed participants, those who linked were significantly younger than those who did not (*t*_1_=3.69, *P*<.001) and were more likely to experience unstable housing (*χ*^2^_1_=15.50, *P*<.001). No other significant associations were observed between exposure or linkage and study covariates (see Table 1 for *P* values). Table 2 presents the raw observed means and proportions of study variables by condition. Participants in the exposed group had significantly higher 30-day retention rates, greater treatment continuance, and more daily doses administered during the first 3, 7, and 30 days after admission (all *P*s>.001).

**Table 1:** Sociodemographics for eligible participants (n=1524).

Sociodemographics		Eligible, n (%)	Exposed (n=1205), n (%)	Control (n=319), n (%)	Pearson chi-square/*t* test^a^ (*df*)	*P* value^b^	Among the exposed		Control/intervention	
							Linked (n=363)	Not linked (n=842)	Pearson chi-square/*t* test (*df*)	*P* value^b^
**Fentanyl-positive on admission**					4.79 (1)	.03			3.27 (1)	.07
	No	365 (24.0)	304 (25.2)	61 (19.1)			105 (28.9)	199 (23.6)		
	Yes	1115 (73.2)	869 (72.1)	246 (77.1)			252 (69.4)	617 (73.3)		
	Unknown	44 (2.9)	32 (2.7)	12 (3.8)			6 (1.7)	26 (3.1)		
**Amphetamine-positive on admission**					1.58 (1)	.21			0.30 (1)	.58
	No	547 (35.9)	443 (36.8)	104 (32.6)			139 (38.3)	304 (36.1)		
	Yes	933 (61.2)	730 (60.6)	203 (63.6)			218 (60.1)	512 (60.8)		
	Unknown	44 (2.9)	32 (2.7)	12 (3.8)			6 (1.7)	26 (3.1)		
**Moderate-to-severe depression**					1.94 (1)	.16			0.23 (1)	.63
	No	1327 (87.1)	1045 (86.7)	289 (90.6)			313 (86.2)	732 (86.9)		
	Yes	165 (10.8)	137 (11.4)	28 (8.8)			44 (12.1)	93 (11.0)		
	Unknown	32 (2.1)	23 (1.9)	2 (0.6)			6 (1.7)	17 (2.0)		
**Moderate-to-severe anxiety**					7.36 (1)	.007			0.27 (1)	.60
	No	1263 (82.9)	986 (81.8)	284 (89.0)			297 (81.8)	689 (81.8)		
	Yes	229 (15.0)	196 (16.3)	33 (10.3)			60 (16.5)	136 (16.2)		
	Unknown	32 (2.1)	23 (1.9)	2 (0.6)			6 (1.7)	17 (2.0)		
**Unemployed on admission**					4.83 (1)	.03			7.61 (1)	.006
	No	507 (33.3)	421 (34.9)	92 (28.8)			148 (40.8)	273 (32.4)		
	Yes	985 (64.6)	761 (63.2)	225 (70.5)			209 (57.6)	552 (65.6)		
	Unknown	32 (2.1)	23 (1.9)	2 (0.6)			6 (1.7)	17 (2.0)		
**Had unstable housing on admission**					0.45 (1)	.50			15.74 (1)	*<.001*
	No	1084 (71.1)	865 (71.8)	226 (70.8)			289 (79.6)	576 (68.4)		
	Yes	408 (26.8)	317 (26.3)	91 (28.5)			68 (18.7)	249 (29.6)		
	Unknown	32 (2.1)	23 (1.9)	2 (0.6)			6 (1.7)	17 (2.0)		
**Gender**					0.63 (1)	.43			8.92 (1)	*.003*
	Man	805 (52.8)	630 (52.3)	175 (54.9)			166 (45.7)	464 (55.1)		
	Woman	712 (46.7)	569 (47.2)	143 (44.8)			195 (53.7)	374 (44.4)		
	Unknown	7 (0.5)	6 (0.5)	1 (0.3)			2 (0.6)	4 (0.5)		
**Race**					8.16 (4)	.09			6.32 (4)	.18
	Black	32 (2.1)	30 (2.5)	2 (0.6)			5 (1.4)	25 (3.0)		
	Hispanic/Latino	58 (3.8)	46 (3.8)	12 (3.8)			18 (5.0)	28 (3.3)		
	Native American/Pacific Islander	329 (21.6)	249 (20.7)	80 (25.1)			69 (19.0)	180 (21.4)		
	Unknown	219 (14.4)	181 (15.0)	38 (11.9)			50 (13.8)	131 (15.6)		
	Non-Hispanic White	886 (58.1)	699 (58.0)	187 (58.6)			221 (60.9)	478 (56.8)		
**Medication type**					1.05 (1)	.31			1.81 (1)	.18
	Methadone	1384 (90.8)	1099 (91.2)	285 (89.3)			325 (89.5)	774 (91.9)		
	Buprenorphine	140 (9.2)	106 (8.8)	34 (10.7)			38 (10.5)	68 (8.1)		
	Age (years) on admission (n=1521), mean (SD)	37.88 (12.4)	37.6 (10.5)	37.7 (10.2)	0.162 (1524)^a^	.87	35.88 (9.193)	38.39 (10.909)	3.82 (1197)^a^	*<.001*

^a^*t* test (unpaired and 2-tailed).

^b^*P* values that remain significant after Benjamini-Hochberg correction for multiple testing are italicized.

**Table 2:** Study variables for eligible participants (n=1524).

Variables		Eligible	Exposed (n=1205)	Control (n=319)	Pearson chi-square/independent samples *t* test (*df*)		*P* value	
**Retention at 30 days, n (%)**						21.64 (1)		<.001
	No	399 (26.2)	283 (23.5)	116 (36.4)				
	Yes	1125 (73.8)	922 (76.5)	203 (63.6)				
**Treatment continuance at 30 days, n (%)**						30.3 (1)		<.001
	No	723 (47.4)	528 (43.8)	195 (61.1)				
	Yes	801 (52.6)	677 (56.2)	124 (38.9)				
**Linked with app at 30 days, n (%)**						N/A^a^		N/A
	No	N/A	842 (69.9)	N/A				
	Yes	N/A	363 (30.1)	N/A				
**Time to loss to follow-up (among lost at 30 days), mean (SD)**		11.15 (8.5)	11.27 (8.52)	10.84 (8.4)	–0.466 (1523)^b^		0.642	
	Time to linkage (among linked at 30 days)	13.1 (8.3)	7.74 (9.06)	N/A	1.461 (1523)^b^		0.145	
	Confirmed doses at 3 days	2.69 (0.6)	2.73 (0.59)	2.56 (0.73)	–4.394 (1523)^b^		<.001	
	Confirmed doses at 7 days	5.71 (1.7)	5.84 (1.63)	5.22 (1.93)	–5.755 (1523)^b^		<.001	
	Confirmed doses at 30 days	20.42 (9.3)	21.19 (1.63)	17.51 (9.8)	–6.373 (1523)^b^		<.001	

^a^N/A: not applicable.

^b^*t* test (unpaired and 2-tailed).

### Exposure

**Figure 3:**
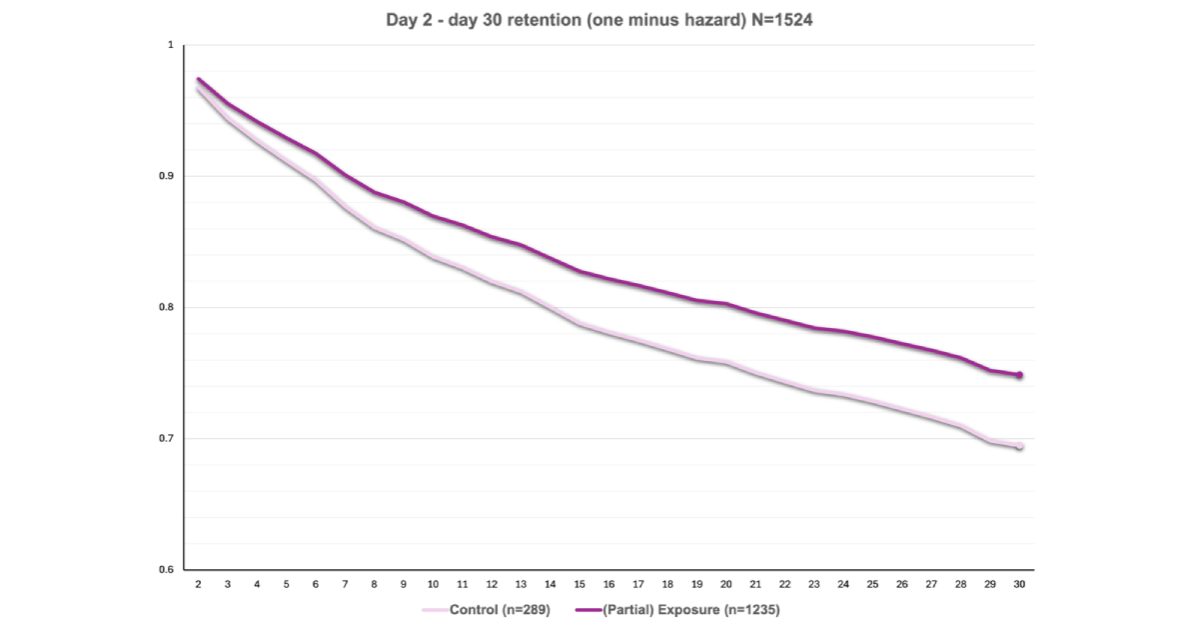
Probability of retention by exposure status. Results are from a cluster-controlled discrete-time survival model estimating daily probability of loss to follow-up as a function of prior-day exposure. The estimated absolute difference in 30-day retention is 74.8% (Exposure) versus 69.5% (Control), yielding a 5.3% improvement.

Results of the exposure analyses are presented in Table 3 and Figure 3. In separate moderator-first models, none of the 11 covariates significantly moderated the effect of exposure (*P*s=.09-.93) after Benjamini-Hochberg correction, indicating that exposure effects were consistent across individual differences within the cohort. In covariate-unadjusted analyses, compared with no exposure, patients whose MHPs were trained in app usage were more likely to be retained at 30 days (hazard ratio [HR] for loss to follow-up 0.80, 95% CI 0.64 to <1.00, *P*=.047), corresponding to an estimated 30-day retention likelihood of 74.8% versus 69.5% among unexposed participants. After covariate adjustment, the direction of the effect remained the same but was no longer statistically significant (HR 0.81, 95% CI 0.65-1.01, *P*=.06).

**Table 3:** Survival model effects of exposure and linkage on subsequent retention.

Model effects		Provider training			Patient linkage						
		All eligibles (n=1524)			All eligibles (n=1524)				Therapist exposed only (n=1209)		
		HR	95% CI	*P* value	HR	95% CI	*P* value	HR		95% CI	*P* value
Covariate-unadjusted exposure (0=provider not trained in the app; 1=provider trained in the app)		0.8	0.64 to <1.00	.047	N/A^a^	N/A	N/A	N/A		N/A	N/A
Covariate-adjusted exposure (0=provider not trained in the app; 1=provider trained in the app)		0.81	0.65 to 1.01	.06	N/A	N/A	N/A	N/A		N/A	N/A
Covariate-unadjusted linkage (0=not linked, 1=linked with the app)		N/A	N/A	N/A	0.63	0.48 to 0.83	.001	0.69		0.50 to 0.95	.02
Covariate-adjusted linkage (0=not linked, 1=linked with the app)		N/A	N/A	N/A	0.65	0.48 to 0.87	.004	0.7		0.51 to 0.97	.03
Fentanyl positive on admission (0=yes, 1=no)		0.91	0.68 to 1.23	.55	0.92	0.68 to 1.25	.60	0.87		0.59 to 1.27	.46
Amphetamine positive on admission		1.51	1.2 to 1.89	<.001	1.51	1.19 to 1.9	.001	1.44		1.07 to 1.94	.01
Moderate-to-severe depression		1.28	0.96 to 1.7	.09	1.28	0.95 to 1.71	.10	1.26		0.87 to 1.84	.22
Moderate-to-severe anxiety		1.04	0.91 to 1.18	.56	1.04	0.93 to 1.16	.47	1.18		0.91 to 1.53	.20
Financial stress		1.37	1.08 to 1.74	.009	1.35	1.06 to 1.72	.01	1.46		1.14 to 1.87	.003
Gender		0.96	0.81 to 1.14	.66	0.98	0.82 to 1.16	.80	0.92		0.78 to 1.09	.34
Age (1=>37+ years, 0=<38 years)		0.81	0.6 to 1.09	.17	0.8	0.6 to 1.06	.12	0.74		0.57 to 0.96	.02
Has had prior admissions (1=yes, 0=no)		0.93	0.67 to 1.29	.67	0.92	0.66 to 1.28	.62	0.94		0.58 to 1.54	.82
Number of prior admissions		1.13	0.99 to 1.28	.06	1.12	0.99 to 1.27	.06	1.11		0.94 to 1.3	.22
**Race/ethnicity (reference non-Hispanic White)**											
	Black or African American	2.34	1.67 to 3.29	<.001	2.2	1.55 to 3.14	<.001	2.36		1.51 to 3.69	<.001
	Hispanic/Latino	1.05	0.67 to 1.64	.84	1.06	0.66 to 1.71	.80	0.84		0.63 to 1.13	.26
	Native American/Pacific Islander	1.37	0.92 to 2.03	.12	1.36	0.9 to 2.06	.15	1.4		0.91 to 2.15	.12
	Unknown	0.96	0.68 to 1.36	.81	0.95	0.67 to 1.34	.76	0.94		0.61 to 1.45	.78
Buprenorphine as MOUD (reference methadone)		2.52	2 to 3.19	<.001	2.55	2.02 to 3.24	<.001	2.78		2.24 to 3.45	<.001

^a^N/A: not applicable.

### Linkage

#### Linkage Analysis Results

**Figure 4:**
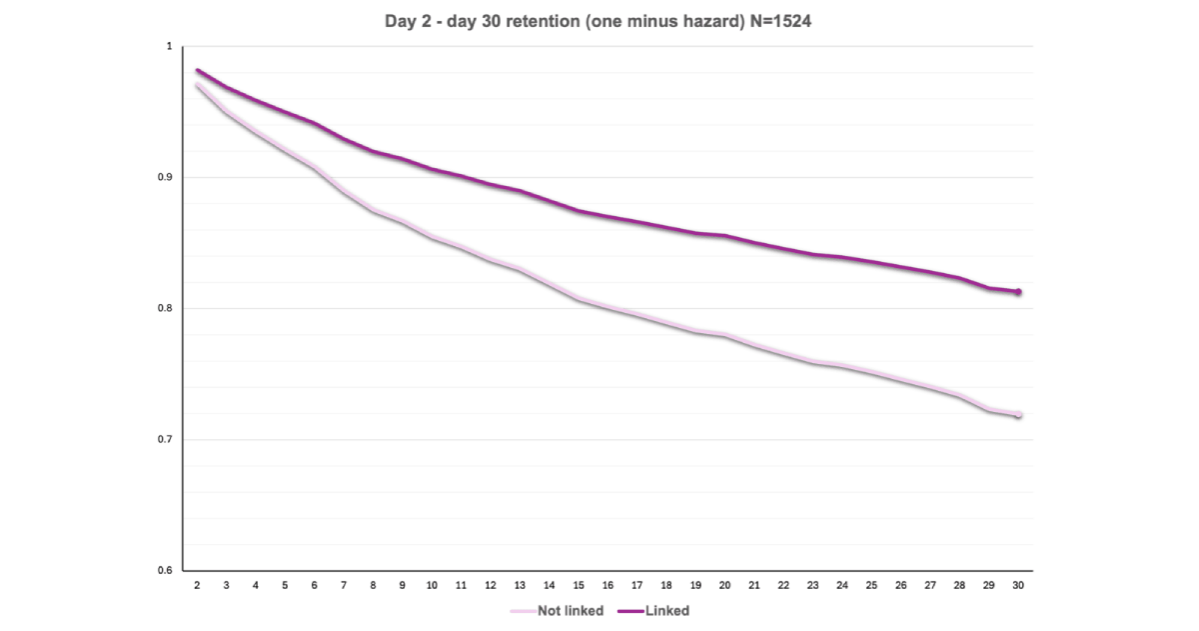
Probability of retention by linkage status. Results are from cluster-controlled discrete-time survival models estimating daily probability of loss to follow-up based on prior-day linkage status in the full sample. The estimated absolute difference in 30-day retention is 81.3% (Linked) versus 72.0% (Not linked), a 9.3% improvement. Hazard ratio for loss to follow-up is 0.63.

Results of the linkage analyses are presented in Table 3 and Figures 4 (across the full sample) and 5 (among the exposed).

**Figure 5:**
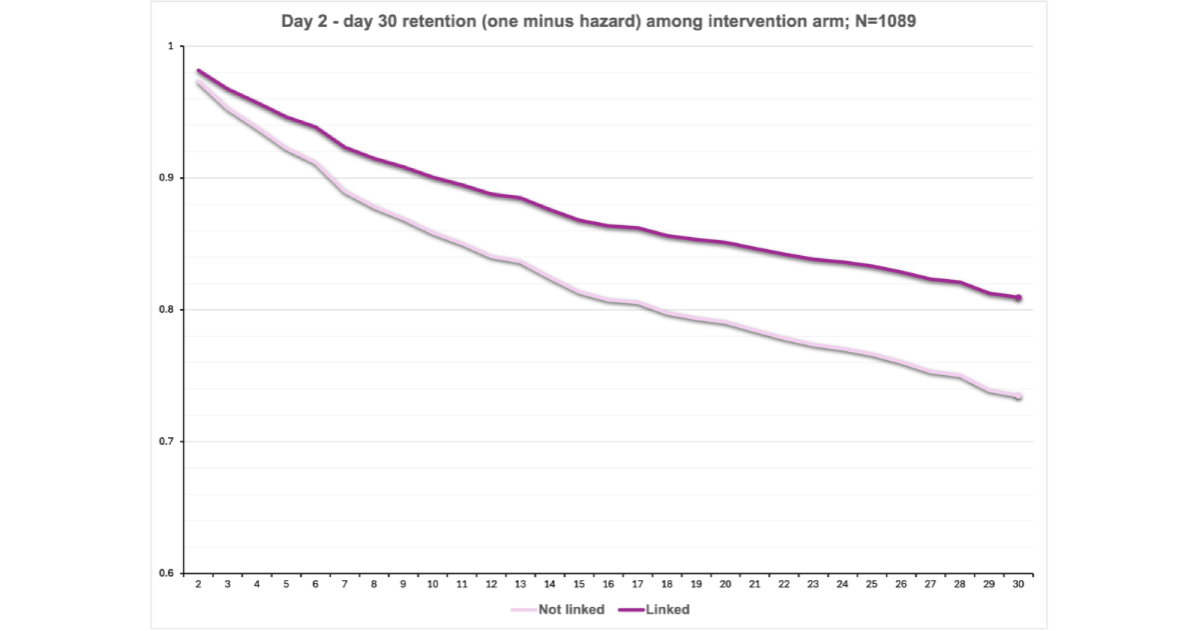
Probability of retention by linkage status. Results are from cluster-controlled discrete-time survival models estimating daily probability of loss to follow-up based on prior-day linkage status among participants in the intervention arm. The estimated absolute difference in 30-day retention is 80.9% (Linked) versus 73.5% (Not linked), a 7.4% improvement. Hazard ratio for loss to follow-up is 0.69.

#### Across the Full Sample (n=1524)

There were no significant interactions between linkage and any covariates (*P*s=.09-.91). In covariate-unadjusted analyses, compared with no linkage, patients who linked were more likely to be retained at 30 days (HR for loss to follow-up 0.63, 95% CI 0.48-0.83, *P*<.001; 81.3% 30-day retention among linked patients vs 72.0% among unlinked patients). Covariate-adjusted effects of linkage were similar in magnitude and remained significant (HR 0.65, 95% CI 0.48-0.87, *P*=.004).

#### Among the Exposed (n=1209)

There were no significant interactions between linkage and any covariates (*P*s=.09-.99) after Benjamini-Hochberg correction. In covariate-unadjusted analyses, compared with no linkage, patients who linked were more likely to be retained at 30 days (HR for loss to follow-up 0.69, 95% CI 0.50-0.95, *P*=.02; 80.9% 30-day retention among linked patients vs 73.5% among unlinked patients). Covariate-adjusted effects of linkage were similar in direction and remained significant (HR 0.70, 95% CI 0.51-0.97, *P*=.03).

### Engagement

Results of engagement analyses are shown in Table 4. There were no significant interactions between engagement and covariates (*P*s=.04-.89) after Benjamini-Hochberg correction. Among patients retained at 1 week (n=1366), minimal, moderate, and high engagement during the first week were each associated with higher 30-day retention compared with no engagement (covariate-unadjusted odds ratios [ORs] 2.79-7.15, *P*s<.001; covariate-adjusted ORs 2.75-6.35, *P*s=.003 to <.001). Additionally, high engagement was associated with greater 30-day retention than minimal engagement in covariate-unadjusted analyses (*b*=–0.941, *P*=.04); this effect was similar in direction but nonsignificant after covariate adjustment (*b*=–0.844, *P*=.07). Among those exposed and retained at 1 week (n=977), minimal, moderate, and high engagement were each associated with higher 30-day retention compared with no engagement (covariate-unadjusted ORs 2.39–6.44, *P*s<.001).

**Table 4:** Links between week 1 patient/clinician engagement and subsequent retention.

Links			Retained at 1 week (n=1366)									Exposed; retained at 1 week (n=977)						
			*b*		*P* value		OR		95% CI		*b*			*P* value		OR		95% CI
**Covariate-unadjusted engagement (reference: 0-1 self-monitoring entries; welcome-message only)**			1.05		N/A^a^		N/A		N/A		1.22			N/A		N/A		N/A
	Minimal engagement (2-4 self-monitoring entries/messages)	1.03		<.001		2.79		1.86-4.18		0.87			<.001		2.39		1.5-3.81	
	Moderate engagement (5-9 self-monitoring entries/messages)	1.52		<.001		4.58		2.68-7.82		1.3			<.001		3.65		1.95-6.82	
	High engagement (10+ self-monitoring entries/messages)	1.97		<.001		7.15		3.1-16.51		1.86			<.001		6.44		2.32-17.92	
**Covariate-adjusted engagement (reference: 0-1 self-monitoring entries; welcome message only)**																		
	Minimal engagement (2-4 self-monitoring entries/messages)	1.01		.003		2.75		1.41-5.34		0.83			.004		2.3		1.31-4.06	
	Moderate engagement (5-9 self-monitoring entries/messages)	1.47		<.001		4.36		2.79-6.82		1.24			<.001		3.45		2-5.94	
	High engagement (10+ self-monitoring entries/messages)	1.85		<.001		6.35		3.08-13.1		1.7			.003		5.45		1.81-16.4	
	Fentanyl-positive on admission (0=yes, 1=no)	0		.99		1		0.66-1.53		0.06			.81		1.06		0.67-1.66	
	Amphetamine-positive on admission	–0.46		.001		0.63		0.48-0.84		–0.33			.15		0.72		0.46-1.12	
	Moderate-to-severe depression	–0.32		.15		0.73		0.48-1.12		–0.22			.38		0.81		0.49-1.31	
	Moderate-to-severe anxiety	0.13		.41		1.13		0.84-1.54		–0.1			.36		0.9		0.72-1.12	
	Financial stress	–0.2		.39		0.82		0.52-1.29		–0.35			.17		0.71		0.43-1.16	
	Gender	0.05		.77		1.05		0.77-1.42		0.14			.37		1.15		0.84-1.58	
	Age (1=>37 years, 0=<38 years)	0.29		.06		1.34		0.99-1.81		0.44			.01		1.55		1.09-2.2	
	Has had prior admissions (1=yes, 0=no)	0.06		.79		1.06		0.68-1.66		–0.08			.79		0.92		0.5-1.69	
	Number of prior admissions	–0.15		.099		0.86		0.72-1.03		–0.13			.19		0.88		0.72-1.07	
**Race/ethnicity (reference: Non-Hispanic White)**																		
	Black or African American	–1		<.001		0.37		0.25-0.55		–1.2			<.001		0.3		0.2-0.46	
	Hispanic/Latino	0.13		.72		1.14		0.56-2.31		0.3			.20		1.35		0.85-2.15	
	Native American/Pacific Islander	–0.3		.32		0.74		0.42-1.33		–0.4			.21		0.67		0.35-1.26	
	Unknown	0.25		.25		1.28		0.84-1.96		0.42			.19		1.52		0.81-2.85	
	Buprenorphine as MOUD (reference: methadone)	–0.33		<.001		0.72		0.66-0.78		–0.38			<.001		0.68		0.62-0.75	

^a^N/A: not applicable.

### Secondary Outcomes

Results are presented in Multimedia Appendix 3. In both covariate-unadjusted and covariate-adjusted models, exposure was associated with a greater likelihood of treatment continuance and with a higher number of MOUD doses taken at 3, 7, and 30 days postintake (covariate-unadjusted *P*s=.007-.037; covariate-adjusted *P*s=.004-.047), among patients retained for at least the corresponding number of days.

Among all eligible patients (n=1378, excluding those whose MHP completed RC training during their ongoing admission), covariate-unadjusted analyses showed that prior linkage predicted a greater likelihood of treatment continuance as well as a higher number of doses at 3, 7, and 30 days (*P*s<.001-.007). After adjusting for covariates, prior linkage remained a significant predictor of treatment continuance, 7-day doses, and 30-day doses (*P*s<.001), but not 3-day doses (*P*=.12).

Among those exposed (n=1089), covariate-unadjusted analyses indicated that prior linkage predicted a greater likelihood of treatment continuance and a higher number of doses at 7 and 30 days (*P*s<.001-.002), but not the number of doses taken at 3 days (*P*=.11). After adjusting for covariates, prior linkage continued to predict greater likelihood of treatment continuance and higher numbers of 7- and 30-day doses (*P*s<.001-.008), but not the number of doses taken at 3 days (*P*=.31).

## Discussion

### Principal Findings

The improvements in 30-day retention and treatment continuance among exposed patients indicate that implementing RC (Recovery Path) can meaningfully enhance standard MOUD care. Based on adjusted model estimates, exposure to the intervention—through MHP training and clinic-level implementation—was associated with a 23% reduction in the hazard of treatment discontinuation (covariate-adjusted HR 0.81).

In the context of MOUD care, this is clinically significant, as retention rates have remained stagnant among the nearly 2.3 million people on MOUD across the United States [21]. Further, among patients whose clinicians were trained, those who linked early via RC showed even higher retention, indicating that downloading the app and linking with their MHP provides benefit beyond exposure alone. In addition, minimal to high engagement during the first week emerged as a strong and consistent predictor of positive outcomes, underscoring the critical importance of early and active engagement. Digital tools such as RC may thus enable proactive, data-informed support during this crucial window, offering a promising avenue for sustaining treatment momentum and preventing early discontinuation.

Our findings align with and extend the work of Kwan et al [10], who conducted a comprehensive meta-analysis evaluating the effectiveness of remote interventions in conjunction with in-person substance use treatment. Supplementing in-person care with remote interventions led to a 39% reduction in the odds of relapse compared with in-person care alone (OR 0.61, 95% CI 0.46-0.81, *P*=.001). Building on this, our study found that patients who linked with an MHP on RC, engaging in a blended care model, had significantly higher treatment retention (81.3%) compared with those not linked (72.0%, *P*<.001). While Kwan et al’s study focused primarily on relapse outcomes, our data show that this synergistic model can also enhance continuity of care in real-world substance use treatment settings. Together, these results underscore the critical role of integrated digital tools in augmenting treatment engagement and supporting sustained recovery.

Secondary analyses also found significant effects for increased daily dosing at 7 and 30 days, but not at 3 days after admission. A likely reason is insufficient sensitivity in the 3-day dose metric to detect meaningful differences in condition effects. The similarity in the valence and amplitude of effects between the 3-day findings and those at 7 and 30 days after admission supports this explanation. Further, a principal intended benefit of linkage via RC is to facilitate greater communication between MHPs and patients; the brief 3-day window may provide a limited opportunity for this effect to fully manifest. Nonetheless, more emphasis can be placed on encouraging MHPs to engage patients and promote app use from day 1 to maximize potential benefits.

Our findings have important implications for clinical practice and health system policy. First, they highlight the value of integrating digital tools not as standalone solutions, but as MHP-augmented enhancements to care delivery. Implementation efforts should prioritize not only MHP training, but also workflow and medical record integration, linkage protocols, and patient onboarding early in the admission process to maximize impact. Second, early engagement data may serve as a practical signal for MHPs and program administrators to identify patients at risk of early treatment discontinuation—offering a basis for real-time, data- and human-driven intervention. At a health system policy level, our findings bolster the case for reimbursement mechanisms and quality metrics that acknowledge the role of digital patient monitoring in OUD treatment. The Centers for Medicare & Medicaid Services have recognized this need by introducing new Healthcare Common Procedure Coding System (HCPCS) codes that facilitate reimbursement for treatment management services related to the patient’s therapeutic use of digital mental health treatment devices [22]. By establishing these codes, the Centers for Medicare & Medicaid Services acknowledges the therapeutic value of integrating digital tools into behavioral health care plans. This policy shift aligns with our study’s demonstration that digitally enhanced blended care models, such as MHP linkage via RC, can significantly improve treatment retention. As MOUD programs work to improve retention and address treatment gaps, these reimbursement pathways may enable the scalable deployment of digital tools that expand care capacity and support long-term recovery across broader populations.

Additionally, given the legitimate concerns recently raised by Stoltman and Terplan [23] regarding privacy violations and the use of surveillance technologies in digital health tools for substance use disorders, our study provides an important counterexample. The intervention examined here was deployed within an IRB-approved protocol, and Recovery Path was developed in accordance with established privacy-by-design principles. Recovery Path strictly avoids third-party surveillance technologies, does not collect or share sensitive health data for commercial purposes, and maintains full compliance with relevant privacy laws, including 42 CFR Part 2 [24] and HIPAA [25]. These practices help mitigate the ethical concerns associated with digital interventions and support the continued development of trustworthy, patient-centered technologies for addiction treatment.

Importantly, to date, adoption of digital tools in OUD treatment has not reached a scale sufficient to meet the needs of many individuals requiring care. Much of the existing research has focused on feasibility or efficacy under ideal conditions, leaving a critical gap in understanding how such tools can be effectively deployed in real-world MOUD treatment settings. One national survey found that only 34% of US health care organizations reported using any category of digital health technology for OUD treatment [26]. This study contributes to bridging that gap, demonstrating that a blended care model—where digital tools integrate into existing clinical workflows and medical record systems—can be implemented across multiple outpatient MOUD settings with measurable improvements in patient retention. Future studies should also examine cost-effectiveness, equity of access, and patient-centered outcomes to further clarify the impact of such interventions.

While the study’s multisite stepped-wedge design provides considerable strengths in terms of power, inferential robustness, and generalizability, several limitations should be noted. First, due to the nature of the app—which facilitates interaction between MHP and patient—there was no obvious method for including an attention-matched control app. The baseline standard of care at each site served as the comparator for evaluating the effects of app training and usage on retention, linkage, and engagement, consistent with the usual stepped-wedge crossover design [27]. Furthermore, for feasibility reasons, the stepped-wedge design implemented site transitions into the experimental condition within short time frames, with each site crossing over 1 week apart. This raises the possibility that unobserved temporal factors could have influenced app adoption. However, exploratory analyses revealed no evidence of secular trends over time in any outcome measures, using either parametric or nonparametric definitions of change. We also focused on short-term outcomes (30 days); future research should examine sustained retention, relapse, and recovery indicators over longer time horizons.

While we were able to demonstrate differences in retention between patients who linked and those who did not, the pragmatic nature of the trial meant that a substantial number of patients did not link after initial exposure. Reasons for this may have varied (eg, lack of a phone during admission, refusal to use the app), potentially introducing bias if these factors were associated with retention. To mitigate this risk, all MHPs followed the same approach with every new patient and made consistent efforts to link all new admissions equally. Future research will examine reasons for late adoption or refusal to engage with the app. Additionally, MHPs have varying levels of education, and although all received the same training both in clinical service delivery and in the use of the app, it is possible that those with greater education or clinical experience may respond differently to RC. Investigating this potential moderation effect is an area for future study.

Patients were identified using a global identifier, ensuring that dosing records remained continuous if they transferred among any of the CMS clinics. The data do not indicate whether a patient newly entering treatment had transferred from an outside clinic and was therefore already established on methadone at entry. To address this, the maximum allowable first-day dose (30 mg before April 2024 and 50 mg thereafter) was used to classify patients as either newly started on methadone or as transfers, since most transfer patients receive doses above these thresholds. Some patients may be misclassified using this approach; however, this is not expected to introduce bias in the stepped-wedge study design. For new patients started on buprenorphine, it was not possible to determine whether they had been taking it outside of CMS before admission. Nevertheless, buprenorphine use was accounted for statistically and represented a minority of the total sample. Regarding ethnic diversity, because patients were drawn from clinics in Alaska, Montana, and North Dakota, the sample included a high representation of Native American/Pacific Islander patients and, correspondingly, a lower representation of Black/African American and Hispanic/Latino participants. These rates deviate from national-level racial and ethnic averages, highlighting the value of future replications of this study in populations with different demographic compositions.

### Conclusions

This study demonstrates that implementing RC—a digital remote patient monitoring app—within real-world outpatient OUD treatment settings can significantly improve patient retention and treatment continuance. The stepped-wedge cluster randomized design provides robust evidence, extending the growing literature on digital interventions in substance use treatment from feasibility studies to measurable clinical impact.

Our findings have direct relevance for health systems and policy makers seeking practical, cost-effective strategies to improve MOUD delivery at scale. As national and local governments continue to confront high overdose rates [28,29] and strained treatment infrastructure, evidence-based digital interventions such as RC offer a timely and impactful opportunity.

Sustained investment in digital infrastructure, implementation science, reimbursement mechanisms, and equitable access will be essential to ensure these tools reach their full potential. Future research should build on this work to examine long-term outcomes, differential effectiveness across populations and varying levels of substance use, and the cost-effectiveness of implementing digital care models in routine practice.

## Appendix

Multimedia Appendix 1 Overview of data transformations and key statistical considerations used in the analysis, along with full code syntax for all analytic procedures.

Multimedia Appendix 2 CONSORT-eHEALTH checklist (V 1.6.1).

Multimedia Appendix 3 Additional analysis.

## Abbreviations

CMS: Community Medical Service
CONSORT: Consolidated Standards of Reporting Trials
HCPCS: Healthcare Common Procedure Coding System
HIPAA: Health Insurance Portability and Accountability Act
HR: hazard ratio
IRB: institutional review board
MHP: mental health professional
MOUD: medication for opioid use disorder
OR: odds ratio
OUD: opioid use disorder
RC: Recovery Connect

## Data Availability

The datasets generated and analyzed during this study are not publicly available due to data confidentiality agreements between Bright Therapeutics and Community Medical Services; however, select deidentified data are available from the corresponding author upon reasonable request.
