# Supplementary material for: Implementation of a Mobile Digital Tool Supporting Medication for Opioid Use Disorder Treatment Improves Retention: Stepped-Wedge Cluster Randomized Controlled Trial
Source: J Med Internet Res. 2025 Dec 22;27:e83346. doi: 10.2196/83346 (PMC12770919; doi:10.2196/83346)
Supplement: Multimedia Appendix 1 [file jmir_v27i1e83346_app1.docx]

MULTIMEDIA APPENDIX

Overview, data transformations, and syntax

**Overview**

For this study, data was available regarding retention on a given day for each patient. However, implementation of the app was administered in wedges across agencies, and administered to clinicians. Therefore, it was possible that over the course of a patient’s admission, the clinic in question entered the implementation phase and became subject to the intervention. For this reason, some patients were subject to partial exposure. In order to accommodate this statistically, we opted to analyze exposure as a time-varying effect predicting next-day retention of the patient. This way, patients who were partially exposed would statistically be under exposure only on those days during which the clinic was in the intervention condition, e.g.

-CLINIC 1 enters intervention on August 15^th^

-PATIENTS 1 AND 2 at CLINIC 1 are admitted on August 1^st^.

-PATIENT 1 is lost to follow-up on August 14^th^.

-PATIENT 2 is not lost to follow-up in the first 30 days.

-Based on these specifications, although patients 1 and 2 started on the same date, one is partially exposed to the intervention, whereas the other is not, owing to having dropped out.

Under normal circumstances in multilevel logistic regression at the patient level, this would result in bias, as patient 2 had more opportunity to be exposed due to remaining in treatment longer. For this reason, we employ non-parametrically-adjusted discrete-time survival modeling (Muthen et al, 2020) with exposure as a time-varying predictor, such that exposure is only ‘1’ for patient 2 on those days following August 15^th^. Prior to this, exposure is ‘0’, meaning that statistically, the participant is treated as not under exposure on those dates. This prevents bias from affecting the effect of exposure in the context of survival analysis.

**Data Transformations**

The following is a complete record of data transformations.

1. Data was drawn from two datasets: one is the composite of all admissions records at participating clinics during the trial period (n=1961). The other is the record of downloading, linking, and engagement with the app for those participants who had at least downloaded the app.
2. Multiple admissions were identified by matching patient IDs. Earlier admissions of the same patient were excluded (243 cases). Later admissions were excluded if app usage had occurred during previous admissions (24 cases). *Note: the presence and amount of earlier admissions was controlled for statistically during the analytical step.*
3. Patients with buprenorphine doses of 50 or greater were excluded, as it was assumed this indicated that the patient was transferred between clinics during an ongoing admission (n=170). This left the set of trial participants (n=1524).
4. For each patient, an exposure date was calculated based on when their participating clinic was first inducted on the intervention.
5. For each patient, a set of 30 variables was created indicating whether they were exposed on a given day during their admission. Any crossover into the intervention was determined based on the difference between the date of first admission and the exposure date. E.g.
   1. Non-exposed patient: 0 0 0 0 0 0 0 0 0 0 0 0 0 0 0 …
   2. Partially-exposed patient: 0 0 0 0 0 0 0 0 0 1 1 1 1 1 1 1..
   3. Fully-exposed patient: 1 1 1 1 1 1 1 1 1 1 1 1 1 1 1.
6. Step 4 was repeated for a separate variable indicating linkage with the app had occurred. Unlike exposure, linkage with the app was not analyzed with the intention of identifying causal effects; it was assumed that both observed and unobserved individual differences may have contributed to linkage.
7. We investigated whether time independently predicted changes in loss to follow-up, controlling for intervention status. The effect of time was both examined continuously and piecewise, and no significant trends in the effect of time were identified.
8. A set of discrete-time survival variables reflecting loss to follow-up as an event were coded. Loss to follow-up was coded as the event (1). Retention was coded as 0. Days subsequent to loss to follow-up were coded as missing data, as per discrete-time survival analysis convention (Singer & Willett). E.g.
   1. Retained patient: 0 0 0 0 0 0 0 … 0 0 0 0 0 (up to day 30)
   2. Lost patient: 0 0 0 0 0 0 1 -99 -99 -99 -99 (where -99 indicates missing data)
9. Transformations to compute covariates were applied. In order to avoid bias and sparse coverage, all covariates were dichotomized prior to analysis.
10. Engagement was coded based on procedures described in the main manuscript. Analysis of engagement was performed via non-parametric standard-error-adjusted logistic regression analyses using a full-information maximum likelihood estimator (Muthen et al, 2020).
11. Data was converted to Mplus format and exported to Mplus.
12. Following are the syntaxes for all unadjusted and adjusted analyses investigating discrete-time survival modeling outcomes:

**Analysis 1: Unadjusted prediction of loss by exposure**

VARIABLE:

NAMES ARE EPClin Exposure0-Exposure29 Lost1-Lost30 Days30 LinkageDate Exposure3;

USEVARIABLES ARE Exposure0-Exposure29 Lost2-Lost30;

missing=all(-99);

cluster is EpClin; *!cluster statement allows a non-parametric adjustment for nesting by clinic*

categorical are lost2-lost30;

ANALYSIS:

type is Complex;

Estimator=MLR; *!MLR indicates full information maximum likelihood with a sandwich estimator for robust standard errors.*

process=6;

MODEL:

f by Lost2-Lost30@1;

f@0;

lost2 on exposure0(a); *!(a) constrained parameter estimates to equality over time.*

lost3 on exposure1(a);

lost4 on exposure2(a);

lost5 on exposure3(a);

lost6 on exposure4(a);

lost7 on exposure5(a);

lost8 on exposure6(a);

lost9 on exposure7(a);

lost10 on exposure8(a);

lost11 on exposure9(a);

lost12 on exposure10(a);

lost13 on exposure11(a);

lost14 on exposure12(a);

lost15 on exposure13(a);

lost16 on exposure14(a);

lost17 on exposure15(a);

lost18 on exposure16(a);

lost19 on exposure17(a);

lost20 on exposure18(a);

lost21 on exposure19(a);

lost22 on exposure20(a);

lost23 on exposure21(a);

lost24 on exposure22(a);

lost25 on exposure23(a);

lost26 on exposure24(a);

lost27 on exposure25(a);

lost28 on exposure26(a);

lost29 on exposure27(a);

lost30 on exposure28(a);

output: sampstat stand;

**Analysis 2: Adjusted** **prediction of loss by exposure**

VARIABLE:

NAMES ARE epclin Exposure0-Exposure29 Lost1-Lost30 Days30 fent amph depr anx UnEither gender dichage MultiEp

EpNo Black Hisp NAPI Unknown drtype

USEVARIABLES ARE Exposure1-Exposure29 Lost2-Lost30

**fent amph depr anx UnEither gender dichage MultiEp EpNo**

**Black Hisp NAPI Unknown drtype**;

missing=all(-99);

cluster is EpClin;

categorical are lost2-lost30;

ANALYSIS:

type is Complex;

model=nocov;

Estimator=MLR;

process=6;

algorithm=integration;

integration=montecarlo (200);

MODEL:

f by Lost2-Lost30@1;

f@0;

**f on fent amph depr anx UnEither gender dichage MultiEp EpNo**

**Black Hisp NAPI Unknown drtype;**

;

[fent depr anx gender Amph];

lost2 on Exposure1 (a1);

lost3 on Exposure2 (a1);

lost4 on Exposure3 (a1);

lost5 on Exposure4 (a1);

lost6 on Exposure5 (a1);

lost7 on Exposure6 (a1);

lost8 on Exposure7 (a1);

lost9 on Exposure8 (a1);

lost10 on Exposure9 (a1);

lost11 on Exposure10 (a1);

lost12 on Exposure11 (a1);

lost13 on Exposure12 (a1);

lost14 on Exposure13 (a1);

lost15 on Exposure14 (a1);

lost16 on Exposure15 (a1);

lost17 on Exposure16 (a1);

lost18 on Exposure17 (a1);

lost19 on Exposure18 (a1);

lost20 on Exposure19 (a1);

lost21 on Exposure20 (a1);

lost22 on Exposure21 (a1);

lost23 on Exposure22 (a1);

lost24 on Exposure23 (a1);

lost25 on Exposure24 (a1);

lost26 on Exposure25 (a1);

lost27 on Exposure26 (a1);

lost28 on Exposure27 (a1);

lost29 on Exposure28 (a1);

lost30 on Exposure29 (a1);

output: sampstat stand;

**Analysis 3: Unadjusted** **prediction of loss by linkage – full sample**

VARIABLE:

NAMES ARE EPClin Link0-Link28 Lost1-Lost30 Days30 LinkageDate Exposure3;

USEVARIABLES ARE Link0-Link28 Lost2-Lost30;

missing=all(-99);

cluster is EpClin; *!cluster statement allows a non-parametric adjustment for nesting by clinic*

categorical are lost2-lost30;

ANALYSIS:

type is Complex;

Estimator=MLR; *!MLR indicates full information maximum likelihood with a sandwich estimator for robust standard errors.*

process=6;

MODEL:

f by Lost2-Lost30@1;

f@0;

lost2 on exposure0(a); *!(a) constrained parameter estimates to equality over time.*

lost3 on exposure1(a);

lost4 on exposure2(a);

lost5 on exposure3(a);

lost6 on exposure4(a);

lost7 on exposure5(a);

lost8 on exposure6(a);

lost9 on exposure7(a);

lost10 on exposure8(a);

lost11 on exposure9(a);

lost12 on exposure10(a);

lost13 on exposure11(a);

lost14 on exposure12(a);

lost15 on exposure13(a);

lost16 on exposure14(a);

lost17 on exposure15(a);

lost18 on exposure16(a);

lost19 on exposure17(a);

lost20 on exposure18(a);

lost21 on exposure19(a);

lost22 on exposure20(a);

lost23 on exposure21(a);

lost24 on exposure22(a);

lost25 on exposure23(a);

lost26 on exposure24(a);

lost27 on exposure25(a);

lost28 on exposure26(a);

lost29 on exposure27(a);

lost30 on exposure28(a);

output: sampstat stand;

**Analysis 4: Unadjusted** **prediction of loss by linkage – Among exposed**

VARIABLE:

NAMES ARE EPClin Exposure0-Exposure29 Lost1-Lost30 Days30 LinkageDate Exposure3;

USEVARIABLES ARE Link0-Link28 Lost2-Lost30;

**SUBPOPULATION ARE Exposure3=1;**

missing=all(-99);

cluster is EpClin; *!cluster statement allows a non-parametric adjustment for nesting by clinic*

categorical are lost2-lost30;

ANALYSIS:

type is Complex;

Estimator=MLR; *!MLR indicates full information maximum likelihood with a sandwich estimator for robust standard errors.*

process=6;

MODEL:

f by Lost2-Lost30@1;

f@0;

lost2 on exposure0(a); *!(a) constrained parameter estimates to equality over time.*

lost3 on exposure1(a);

lost4 on exposure2(a);

lost5 on exposure3(a);

lost6 on exposure4(a);

lost7 on exposure5(a);

lost8 on exposure6(a);

lost9 on exposure7(a);

lost10 on exposure8(a);

lost11 on exposure9(a);

lost12 on exposure10(a);

lost13 on exposure11(a);

lost14 on exposure12(a);

lost15 on exposure13(a);

lost16 on exposure14(a);

lost17 on exposure15(a);

lost18 on exposure16(a);

lost19 on exposure17(a);

lost20 on exposure18(a);

lost21 on exposure19(a);

lost22 on exposure20(a);

lost23 on exposure21(a);

lost24 on exposure22(a);

lost25 on exposure23(a);

lost26 on exposure24(a);

lost27 on exposure25(a);

lost28 on exposure26(a);

lost29 on exposure27(a);

lost30 on exposure28(a);

output: sampstat stand;

**Analysis 5: Adjusted** **prediction of loss by linkage – full sample**

VARIABLE:

NAMES ARE epclin Link0-Link29 Lost1-Lost30 Days30 fent amph depr anx UnEither gender dichage MultiEp

EpNo Black Hisp NAPI Unknown drtype

USEVARIABLES ARE Link1-Link29 Lost2-Lost30

**fent amph depr anx UnEither gender dichage MultiEp EpNo**

**Black Hisp NAPI Unknown drtype**;

missing=all(-99);

cluster is EpClin;

categorical are lost2-lost30;

ANALYSIS:

type is Complex;

model=nocov;

Estimator=MLR;

process=6;

algorithm=integration;

integration=montecarlo (200);

MODEL:

f by Lost2-Lost30@1;

f@0;

**f on fent amph depr anx UnEither gender dichage MultiEp EpNo**

**Black Hisp NAPI Unknown drtype;**

;

[fent depr anx gender Amph];

lost2 on Link1 (a1);

lost3 on Link2 (a1);

lost4 on Link3 (a1);

lost5 on Link4 (a1);

lost6 on Link5 (a1);

lost7 on Link6 (a1);

lost8 on Link7 (a1);

lost9 on Link8 (a1);

lost10 on Link9 (a1);

lost11 on Link10 (a1);

lost12 on Link11 (a1);

lost13 on Link12 (a1);

lost14 on Link13 (a1);

lost15 on Link14 (a1);

lost16 on Link15 (a1);

lost17 on Link16 (a1);

lost18 on Link17 (a1);

lost19 on Link18 (a1);

lost20 on Link19 (a1);

lost21 on Link20 (a1);

lost22 on Link21 (a1);

lost23 on Link22 (a1);

lost24 on Link23 (a1);

lost25 on Link24 (a1);

lost26 on Link25 (a1);

lost27 on Link26 (a1);

lost28 on Link27 (a1);

lost29 on Link28 (a1);

lost30 on Link29 (a1);

output: sampstat stand;

**Analysis 6: Adjusted** **prediction of loss by linkage – Among exposed**

VARIABLE:

NAMES ARE epclin Link0-Link29 Lost1-Lost30 Days30 fent amph depr anx UnEither gender dichage MultiEp

EpNo Black Hisp NAPI Unknown drtype;

**SUBPOPULATION ARE Exposure3=1;**

USEVARIABLES ARE Link1-Link29 Lost2-Lost30

**fent amph depr anx UnEither gender dichage MultiEp EpNo**

**Black Hisp NAPI Unknown drtype**;

missing=all(-99);

cluster is EpClin;

categorical are lost2-lost30;

ANALYSIS:

type is Complex;

model=nocov;

Estimator=MLR;

process=6;

algorithm=integration;

integration=montecarlo (200);

MODEL:

f by Lost2-Lost30@1;

f@0;

**f on fent amph depr anx UnEither gender dichage MultiEp EpNo**

**Black Hisp NAPI Unknown drtype;**

;

[fent depr anx gender Amph];

lost2 on Link1 (a1);

lost3 on Link2 (a1);

lost4 on Link3 (a1);

lost5 on Link4 (a1);

lost6 on Link5 (a1);

lost7 on Link6 (a1);

lost8 on Link7 (a1);

lost9 on Link8 (a1);

lost10 on Link9 (a1);

lost11 on Link10 (a1);

lost12 on Link11 (a1);

lost13 on Link12 (a1);

lost14 on Link13 (a1);

lost15 on Link14 (a1);

lost16 on Link15 (a1);

lost17 on Link16 (a1);

lost18 on Link17 (a1);

lost19 on Link18 (a1);

lost20 on Link19 (a1);

lost21 on Link20 (a1);

lost22 on Link21 (a1);

lost23 on Link22 (a1);

lost24 on Link23 (a1);

lost25 on Link24 (a1);

lost26 on Link25 (a1);

lost27 on Link26 (a1);

lost28 on Link27 (a1);

lost29 on Link28 (a1);

lost30 on Link29 (a1);

output: sampstat stand;

**Analysis 7-11: Prediction of Secondary Treatment Outcomes**

VARIABLE:

NAMES ARE EPClin **TC30 EPCR30 EPCR7 EPCR3** Exposure3 MultiEp Epno

Fent Amph Depr Anx Unemp UnstH Gender Race Age Days30New DrType

linkage30 Linkage3 Linkage7;

USEVARIABLES ARE EPCR30 linkage30

!MultiEp EpNo Amph

!fent depr anx gender DrType

!Black Hisp NAPI Unknown dichage UnEither *!This part uncommented for adjusted analyses*

;

SUBPOPULATION are Exposure3 NE 2; *!Excludes partial exposure*

!categorical is TC30; *!This part uncommented for categorical outcome (TC30)*

missing=all(-99);

cluster is EpClin;

ANALYSIS:

type is Complex;

model=nocov;

Estimator=MLR;

process=6;

MODEL:

EPCR30 on linkage30; *!Repeated in separate analyses for* ***TC30 EPCR30 EPCR7*** ***EPCR3***

!EPCR30 on linkage30 fent amph depr anx UnEither gender dichage MultiEp EpNo

! Black Hisp NAPI Unknown drtype; *!This part uncommented for adjusted analyses*

output: sampstat stand;
