# Supplementary material for: Implementation of a Mobile Digital Tool Supporting Medication for Opioid Use Disorder Treatment Improves Retention: Stepped-Wedge Cluster Randomized Controlled Trial
Source: J Med Internet Res. 2025 Dec 22;27:e83346. doi: 10.2196/83346 (PMC12770919; doi:10.2196/83346)
Supplement: Multimedia Appendix 2 [file jmir_v27i1e83346_app2.pdf]

CONSORT-EHEALTH (V 1.6.1) - Submission/Publication Form

The CONSORT-EHEALTH checklist is intended for authors of randomized trials evaluating web-based and Internet-based applications/interventions, including mobile interventions, electronic games (incl multiplayer games), social media, certain telehealth applications, and other interactive and/or networked electronic applications. Some of the items (e.g. all subitems under item 5 - description of the intervention) may also be applicable for other study designs.

The goal of the CONSORT EHEALTH checklist and guideline is to be
a) a guide for reporting for authors of RCTs,
b) to form a basis for appraisal of an ehealth trial (in terms of validity)

CONSORT-EHEALTH items/subitems are MANDATORY reporting items for studies published in the Journal of Medical Internet Research and other journals / scientific societies endorsing the checklist.

Items numbered 1., 2., 3., 4a., 4b etc are original CONSORT or CONSORT-NPT (non-pharmacologic treatment) items.
Items with Roman numerals (i., ii, iii, iv etc.) are CONSORT-EHEALTH extensions/clarifications.

As the CONSORT-EHEALTH checklist is still considered in a formative stage, we would ask that you also RATE ON A SCALE OF 1-5 how important/useful you feel each item is FOR THE PURPOSE OF THE CHECKLIST and reporting guideline (optional).

Mandatory reporting items are marked with a red *.
In the textboxes, either copy & paste the relevant sections from your manuscript into this form - please include any quotes from your manuscript in QUOTATION MARKS,
or answer directly by providing additional information not in the manuscript, or elaborating on why the item was not relevant for this study.

YOUR ANSWERS WILL BE PUBLISHED AS A SUPPLEMENTARY FILE TO YOUR PUBLICATION IN JMIR AND ARE CONSIDERED PART OF YOUR PUBLICATION (IF ACCEPTED).
Please fill in these questions diligently. Information will not be copyedited, so please use proper spelling and grammar, use correct capitalization, and avoid abbreviations.

DO NOT FORGET TO SAVE AS PDF _AND_ CLICK THE SUBMIT BUTTON SO YOUR ANSWERS ARE IN OUR DATABASE !!!

Citation Suggestion (if you append the pdf as Appendix we suggest to cite this paper in the caption):
Eysenbach G, CONSORT-EHEALTH Group

CONSORT-EHEALTH: Improving and Standardizing Evaluation Reports of Web-based and Mobile Health Interventions
J Med Internet Res 2011;13(4):e126
URL: http://www.jmir.org/2011/4/e126/
doi: 10.2196/jmir.1923
PMID: 22209829

jorge.palacios@brighttherapeutics.com
Switch account

Draft not saved

Not shared

* Indicates required question

Your name *

First Last

Jorge Palacios

Primary Affiliation (short), City, Country *

University of Toronto, Toronto, Canada

Bright Therapeutics, San Francisco, USA

Your e-mail address *

abc@gmail.com

jorge.palacios@brighttherapeutics.com

Title of your manuscript *

Provide the (draft) title of your manuscript.

Implementation of Digital Tools Supporting MOUD Treatment Improves Retention: A Stepped-Wedge Cluster Randomized Trial

Name of your App/Software/Intervention *

If there is a short and a long/alternate name, write the short name first and add the long name in brackets.

Recovery Connect (white-labelled version of Re

Evaluated Version (if any)

e.g. "V1", "Release 2017-03-01", "Version 2.0.27913"

Android version 1.2.3; iOS version 1.3.8

Language(s) *

What language is the intervention/app in? If multiple languages are available, separate by comma (e.g. "English, French")

English

URL of your Intervention Website or App

e.g. a direct link to the mobile app on app in appstore (itunes, Google Play), or URL of the website. If the intervention is a DVD or hardware, you can also link to an Amazon page.

https://play.google.com/store/apps/details?id=app.recoveryjourney.patient&hl=en_US

URL of an image/screenshot (optional)

https://share.google/images/WNCgE4ESYu1EfX0yI

Accessibility *

Can an enduser access the intervention presently?

- ○ access is free and open
- ◉ access only for special usergroups, not open
- ○ access is open to everyone, but requires payment/subscription/in-app purchases
- ○ app/intervention no longer accessible
- ○ Other:

Primary Medical Indication/Disease/Condition *

e.g. "Stress", "Diabetes", or define the target group in brackets after the condition, e.g. "Autism (Parents of children with)", "Alzheimers (Informal Caregivers of)"

Opioid Use Disorder

Primary Outcomes measured in trial *

comma-separated list of primary outcomes reported in the trial

Treatment retention

Secondary/other outcomes

Are there any other outcomes the intervention is expected to affect?

Treatment continuance, 3, 7, and 30 day doses

Recommended "Dose" *

What do the instructions for users say on how often the app should be used?

- ○ Approximately Daily
- ○ Approximately Weekly
- ○ Approximately Monthly
- ○ Approximately Yearly
- ○ "as needed"
- ● Other: No instructions specifically say to use for X amount, however users m

Approx. Percentage of Users (starters) still using the app as recommended after 3 months *

- (selected) unknown / not evaluated
- 0-10%
- 11-20%
- 21-30%
- 31-40%
- 41-50%
- 51-60%
- 61-70%
- 71%-80%
- 81-90%
- 91-100%
- Other:

Overall, was the app/intervention effective? *

- yes: all primary outcomes were significantly better in intervention group vs control
- (selected) partly: SOME primary outcomes were significantly better in intervention group vs control
- no statistically significant difference between control and intervention
- potentially harmful: control was significantly better than intervention in one or more outcomes
- inconclusive: more research is needed
- Other:

Article Preparation Status/Stage *

At which stage in your article preparation are you currently (at the time you fill in this form)

- ○ not submitted yet - in early draft status
- ○ not submitted yet - in late draft status, just before submission
- ◉ submitted to a journal but not reviewed yet
- ○ submitted to a journal and after receiving initial reviewer comments
- ○ submitted to a journal and accepted, but not published yet
- ○ published
- ○ Other:

Journal *

If you already know where you will submit this paper (or if it is already submitted), please provide the journal name (if it is not JMIR, provide the journal name under "other")

- ○ not submitted yet / unclear where I will submit this
- ◉ Journal of Medical Internet Research (JMIR)
- ○ JMIR mHealth and UHealth
- ○ JMIR Serious Games
- ○ JMIR Mental Health
- ○ JMIR Public Health
- ○ JMIR Formative Research
- ○ Other JMIR sister journal
- ○ Other:

Is this a full powered effectiveness trial or a pilot/feasibility trial? *

- ○ Pilot/feasibility
- ◉ Fully powered

Manuscript tracking number *

If this is a JMIR submission, please provide the manuscript tracking number under "other" (The ms tracking number can be found in the submission acknowledgement email, or when you login as author in JMIR. If the paper is already published in JMIR, then the ms tracking number is the four-digit number at the end of the DOI, to be found at the bottom of each published article in JMIR)

- ○ no ms number (yet) / not (yet) submitted to / published in JMIR
- ◉ Other: JMIR ms#83346

TITLE AND ABSTRACT

1a) TITLE: Identification as a randomized trial in the title

1a) Does your paper address CONSORT item 1a? *

I.e does the title contain the phrase "Randomized Controlled Trial"? (if not, explain the reason under "other")

- ◉ yes
- ○ Other:

1a-i) Identify the mode of delivery in the title

Identify the mode of delivery. Preferably use “web-based” and/or “mobile” and/or “electronic game” in the title. Avoid ambiguous terms like “online”, “virtual”, “interactive”. Use “Internet-based” only if Intervention includes non-web-based Internet components (e.g. email), use “computer-based” or “electronic” only if offline products are used. Use “virtual” only in the context of “virtual reality” (3-D worlds). Use “online” only in the context of “online support groups”. Complement or substitute product names with broader terms for the class of products (such as “mobile” or “smart phone” instead of “iphone”), especially if the application runs on different platforms.

	1	2	3	4	5	
subitem not at all important	○	○	○	◉	○	essential

Clear selection

Does your paper address subitem 1a-i? *

Copy and paste relevant sections from manuscript title (include quotes in quotation marks "like this" to indicate direct quotes from your manuscript), or elaborate on this item by providing additional information not in the ms, or briefly explain why the item is not applicable/relevant for your study

"Mobile Digital Tool"

Your answer must have a minimum of 25 characters.

1a-ii) Non-web-based components or important co-interventions in title

Mention non-web-based components or important co-interventions in title, if any (e.g., “with telephone support”).

	1	2	3	4	5	
subitem not at all important	○	○	◉	○	○	essential

Clear selection

Does your paper address subitem 1a-ii?

Copy and paste relevant sections from manuscript title (include quotes in quotation marks "like this" to indicate direct quotes from your manuscript), or elaborate on this item by providing additional information not in the ms, or briefly explain why the item is not applicable/relevant for your study

"Supporting MOUD Treatment"

1a-iii) Primary condition or target group in the title

Mention primary condition or target group in the title, if any (e.g., “for children with Type I Diabetes”) Example: A Web-based and Mobile Intervention with Telephone Support for Children with Type I Diabetes: Randomized Controlled Trial

	1	2	3	4	5	
subitem not at all important	○	○	◉	○	○	essential

Clear selection

Does your paper address subitem 1a-iii? *

Copy and paste relevant sections from manuscript title (include quotes in quotation marks "like this" to indicate direct quotes from your manuscript), or elaborate on this item by providing additional information not in the ms, or briefly explain why the item is not applicable/relevant for your study

Title mentions "MOUD Treatment" although it does not specifically say for people with OUD (Opioid Use Disorder) since it would be redundant

1b) ABSTRACT: Structured summary of trial design, methods, results, and conclusions

NPT extension: Description of experimental treatment, comparator, care providers, centers, and blinding status.

1b-i) Key features/functionalities/components of the intervention and comparator in the METHODS section of the ABSTRACT

Mention key features/functionalities/components of the intervention and comparator in the abstract. If possible, also mention theories and principles used for designing the site. Keep in mind the needs of systematic reviewers and indexers by including important synonyms. (Note: Only report in the abstract what the main paper is reporting. If this information is missing from the main body of text, consider adding it)

	1	2	3	4	5	
subitem not at all important	○	○	○	◉	○	essential

Clear selection

Does your paper address subitem 1b-i? *

Copy and paste relevant sections from the manuscript abstract (include quotes in quotation marks "like this" to indicate direct quotes from your manuscript), or elaborate on this item by providing additional information not in the ms, or briefly explain why the item is not applicable/relevant for your study

"Recovery Connect, which provided real-time monitoring, psychoeducational and skill based learning content, and messaging between patients and clinicians"

1b-ii) Level of human involvement in the METHODS section of the ABSTRACT

Clarify the level of human involvement in the abstract, e.g., use phrases like “fully automated” vs. “therapist/nurse/care provider/physician-assisted” (mention number and expertise of providers involved, if any). (Note: Only report in the abstract what the main paper is reporting. If this information is missing from the main body of text, consider adding it)

	1	2	3	4	5	
subitem not at all important	○	○	◉	○	○	essential

Clear selection

Does your paper address subitem 1b-ii?

Copy and paste relevant sections from the manuscript abstract (include quotes in quotation marks "like this" to indicate direct quotes from your manuscript), or elaborate on this item by providing additional information not in the ms, or briefly explain why the item is not applicable/relevant for your study

"Clusters were sequentially graduated from usual care to a digitally-enhanced model incorporating Recovery Connect"

1b-iii) Open vs. closed, web-based (self-assessment) vs. face-to-face assessments in the METHODS section of the ABSTRACT

Mention how participants were recruited (online vs. offline), e.g., from an open access website or from a clinic or a closed online user group (closed usergroup trial), and clarify if this was a purely web-based trial, or there were face-to-face components (as part of the intervention or for assessment). Clearly say if outcomes were self-assessed through questionnaires (as common in web-based trials). Note: In traditional offline trials, an open trial (open-label trial) is a type of clinical trial in which both the researchers and participants know which treatment is being administered. To avoid confusion, use “blinded” or “unblinded” to indicated the level of blinding instead of “open”, as “open” in web-based trials usually refers to “open access” (i.e. participants can self-enrol). (Note: Only report in the abstract what the main paper is reporting. If this information is missing from the main body of text, consider adding it)

	1	2	3	4	5	
subitem not at all important	○	○	○	●	○	essential

Clear selection

Does your paper address subitem 1b-iii?

Copy and paste relevant sections from the manuscript abstract (include quotes in quotation marks "like this" to indicate direct quotes from your manuscript), or elaborate on this item by providing additional information not in the ms, or briefly explain why the item is not applicable/relevant for your study

"A stepped-wedge cluster randomized trial was conducted across nine outpatient MOUD clinics organized into eight clusters. Clusters were sequentially graduated from usual care to a digitally-enhanced model incorporating Recovery Connect". Given this is a stepped wedge trial conducted in real-word settings, the digital intervention was sequentially implemented and there was no recruitment involved.

1b-iv) RESULTS section in abstract must contain use data

Report number of participants enrolled/assessed in each group, the use/uptake of the intervention (e.g., attrition/adherence metrics, use over time, number of logins etc.), in addition to primary/secondary outcomes. (Note: Only report in the abstract what the main paper is reporting. If this information is missing from the main body of text, consider adding it)

	1	2	3	4	5	
subitem not at all important	○	○	◉	○	○	essential

Clear selection

Does your paper address subitem 1b-iv?

Copy and paste relevant sections from the manuscript abstract (include quotes in quotation marks "like this" to indicate direct quotes from your manuscript), or elaborate on this item by providing additional information not in the ms, or briefly explain why the item is not applicable/relevant for your study

"Patients admitted to clinics which had implemented the app (n = 1205) saw increased retention (74.8%) compared to those that had not (n = 319) (69.5%; p=.047)." We did not report use over time since this was beyond the scope of the study. Engagement was operationalized with what we consider to be meaningful engagement metrics (not just logins) and this is explained in Methods section of the ms.

1b-v) CONCLUSIONS/DISCUSSION in abstract for negative trials

Conclusions/Discussions in abstract for negative trials: Discuss the primary outcome - if the trial is negative (primary outcome not changed), and the intervention was not used, discuss whether negative results are attributable to lack of uptake and discuss reasons. (Note: Only report in the abstract what the main paper is reporting. If this information is missing from the main body of text, consider adding it)

	1	2	3	4	5	
subitem not at all important	○	○	○	●	○	essential

Clear selection

Does your paper address subitem 1b-v?

Copy and paste relevant sections from the manuscript abstract (include quotes in quotation marks "like this" to indicate direct quotes from your manuscript), or elaborate on this item by providing additional information not in the ms, or briefly explain why the item is not applicable/relevant for your study

N/A - trial was positive

INTRODUCTION

2a) In INTRODUCTION: Scientific background and explanation of rationale

2a-i) Problem and the type of system/solution

Describe the problem and the type of system/solution that is object of the study: intended as stand-alone intervention vs. incorporated in broader health care program? Intended for a particular patient population? Goals of the intervention, e.g., being more cost-effective to other interventions, replace or complement other solutions? (Note: Details about the intervention are provided in “Methods” under 5)

	1	2	3	4	5	
subitem not at all important	○	○	○	◉	○	essential

Clear selection

Does your paper address subitem 2a-i? *

Copy and paste relevant sections from the manuscript (include quotes in quotation marks "like this" to indicate direct quotes from your manuscript), or elaborate on this item by providing additional information not in the ms, or briefly explain why the item is not applicable/relevant for your study

"Opioid use disorder (OUD) remains a significant public health challenge, characterized by high morbidity, mortality, and substantial societal costs. Medications for opioid use disorder (MOUD) have been shown to reduce opioid use and mortality by more than 50%, while improving social and health outcomes. Yet, despite this efficacy, MOUD retention remains low, with structural and systemic barriers—such as access to care, treatment setting, and referral source—compounding individual patient factors (availability of treatment, personalization, and motivation) in contributing to high rates of discontinuation"

2a-ii) Scientific background, rationale: What is known about the (type of) system

Scientific background, rationale: What is known about the (type of) system that is the object of the study (be sure to discuss the use of similar systems for other conditions/diagnoses, if appropiate), motivation for the study, i.e. what are the reasons for and what is the context for this specific study, from which stakeholder viewpoint is the study performed, potential impact of findings [2]. Briefly justify the choice of the comparator.

	1	2	3	4	5	
subitem not at all important	○	○	○	◉	○	essential

Clear selection

Does your paper address subitem 2a-ii? *

Copy and paste relevant sections from the manuscript (include quotes in quotation marks "like this" to indicate direct quotes from your manuscript), or elaborate on this item by providing additional information not in the ms, or briefly explain why the item is not applicable/relevant for your study

"Digital interventions hold promise for overcoming common barriers such as stigma, limited clinic access, and resource constraints. A recent systematic review highlights the potential of digital and remote interventions to augment traditional substance use treatment, reducing risk of relapse and offering interactive contact between patients and clinicians. These technologies may not only facilitate improved retention but also foster patient autonomy and continuous engagement with care. "
"Preliminary findings from a large-scale feasibility study indicate that Recovery Connect (RC), a digital remote patient monitoring app, successfully facilitated patient-clinician interactions, was well-accepted, and was associated with improved 30-day retention. However, robust randomized trial evidence evaluating app-based tools in real-world opioid treatment settings remains limited. The current study addresses this gap by rigorously testing the effectiveness of RC implementation through a stepped-wedge cluster randomized design."

2b) In INTRODUCTION: Specific objectives or hypotheses

Does your paper address CONSORT subitem 2b? *

Copy and paste relevant sections from the manuscript (include quotes in quotation marks "like this" to indicate direct quotes from your manuscript), or elaborate on this item by providing additional information not in the ms, or briefly explain why the item is not applicable/relevant for your study

"The main objectives of this study were (1) to assess the effect on retention of implementing RC in outpatient OUD clinics, relative to care as usual, (2) to explore the impact on retention of clinician-patient linkage facilitated by the app, and (3) to examine the association between patient engagement levels and treatment retention"

METHODS

3a) Description of trial design (such as parallel, factorial) including allocation ratio

Does your paper address CONSORT subitem 3a? *

Copy and paste relevant sections from the manuscript (include quotes in quotation marks "like this" to indicate direct quotes from your manuscript), or elaborate on this item by providing additional information not in the ms, or briefly explain why the item is not applicable/relevant for your study

"We conducted a stepped-wedge cluster randomized trial to evaluate the impact of RC on retention and treatment continuance among individuals initiating medication for opioid use disorder (MOUD). This design allowed for sequential implementation of the intervention, such that each cluster transitioned from a control period (usual care) to an intervention period (RC), serving as its own control. Clusters were randomized to crossover into the intervention condition at weekly intervals beginning February 26, 2024, through April 16, 2024. Figure 1 shows the full data collection period alongside dates for crossover at each clinic. The stepped-wedge approach was selected to facilitate pragmatic implementation, reflecting real-world conditions, whilst preserving the ability to evaluate causal effects. This design also allowed us to account for temporal trends and clinic-level variability through staggered rollout."

3b) Important changes to methods after trial commencement (such as eligibility criteria), with reasons

Does your paper address CONSORT subitem 3b? *

Copy and paste relevant sections from the manuscript (include quotes in quotation marks "like this" to indicate direct quotes from your manuscript), or elaborate on this item by providing additional information not in the ms, or briefly explain why the item is not applicable/relevant for your study

N/A - no important changes to methods were made after trial commencement

3b-i) Bug fixes, Downtimes, Content Changes

Bug fixes, Downtimes, Content Changes: ehealth systems are often dynamic systems. A description of changes to methods therefore also includes important changes made on the intervention or comparator during the trial (e.g., major bug fixes or changes in the functionality or content) (5-iii) and other “unexpected events” that may have influenced study design such as staff changes, system failures/downtimes, etc. [2].

	1	2	3	4	5	
subitem not at all important	○	○	○	●	○	essential

Clear selection

Does your paper address subitem 3b-i?

Copy and paste relevant sections from the manuscript (include quotes in quotation marks "like this" to indicate direct quotes from your manuscript), or elaborate on this item by providing additional information not in the ms, or briefly explain why the item is not applicable/relevant for your study

N/A - no major bug fixes, downtimes or content changes occurred during the trial that would influence the methods or results

4a) Eligibility criteria for participants

Does your paper address CONSORT subitem 4a? *

Copy and paste relevant sections from the manuscript (include quotes in quotation marks "like this" to indicate direct quotes from your manuscript), or elaborate on this item by providing additional information not in the ms, or briefly explain why the item is not applicable/relevant for your study

"Eligible participants were adults (18+) newly admitted to treatment between January 1st and November 1st, 2024. Patients were excluded if their dosing suggested a transfer from a non-CMS clinic, or if they had previously used RC before their most recent admission during the study."

4a-i) Computer / Internet literacy

Computer / Internet literacy is often an implicit "de facto" eligibility criterion - this should be explicitly clarified.

	1	2	3	4	5	
subitem not at all important	○	○	◉	○	○	essential

Clear selection

Does your paper address subitem 4a-i?

Copy and paste relevant sections from the manuscript (include quotes in quotation marks "like this" to indicate direct quotes from your manuscript), or elaborate on this item by providing additional information not in the ms, or briefly explain why the item is not applicable/relevant for your study

N/A - since this was a pragmatic, real-world study where we studied the effects of the implementation and use of the app and included all new admissions, except for transfers or prior users of the app, we did not specifically include internet literacy as an inclusion/exclusion criteria.

4a-ii) Open vs. closed, web-based vs. face-to-face assessments:

Open vs. closed, web-based vs. face-to-face assessments: Mention how participants were recruited (online vs. offline), e.g., from an open access website or from a clinic, and clarify if this was a purely web-based trial, or there were face-to-face components (as part of the intervention or for assessment), i.e., to what degree got the study team to know the participant. In online-only trials, clarify if participants were quasi-anonymous and whether having multiple identities was possible or whether technical or logistical measures (e.g., cookies, email confirmation, phone calls) were used to detect/prevent these.

	1	2	3	4	5	
subitem not at all important	○	○	○	◉	○	essential

Clear selection

Does your paper address subitem 4a-ii? *

Copy and paste relevant sections from the manuscript (include quotes in quotation marks "like this" to indicate direct quotes from your manuscript), or elaborate on this item by providing additional information not in the ms, or briefly explain why the item is not applicable/relevant for your study

"Eligible participants were adults (18+) newly admitted to treatment between January 1st and November 1st, 2024."
"Exposure groups were defined by implementation timing and patient app download / linkage to the MHP app."
"During the control period, all clinics followed standard MOUD protocols."
"The implementation of the Recovery Connect app was part of the rollout at participating clinics and integrated into existing treatment pathways."

4a-iii) Information giving during recruitment

Information given during recruitment. Specify how participants were briefed for recruitment and in the informed consent procedures (e.g., publish the informed consent documentation as appendix, see also item X26), as this information may have an effect on user self-selection, user expectation and may also bias results.

	1	2	3	4	5	
subitem not at all important	○	○	○	◉	○	essential

Clear selection

Does your paper address subitem 4a-iii?

Copy and paste relevant sections from the manuscript (include quotes in quotation marks "like this" to indicate direct quotes from your manuscript), or elaborate on this item by providing additional information not in the ms, or briefly explain why the item is not applicable/relevant for your study

"Clinicians signed informed consent to participate in the study. Patients give consent for their anonymized data to be used for research purposes as part of their treatment contract and thus a waiver for consent was approved by the IRB"

4b) Settings and locations where the data were collected

Does your paper address CONSORT subitem 4b? *

Copy and paste relevant sections from the manuscript (include quotes in quotation marks "like this" to indicate direct quotes from your manuscript), or elaborate on this item by providing additional information not in the ms, or briefly explain why the item is not applicable/relevant for your study

"The study was conducted across nine outpatient MOUD clinics operated by Community Medical Services (CMS), a network of Opioid Treatment Programs (OTPs) serving urban and rural populations across multiple U.S. states. Participating clinics were located in Montana (Billings, Bozeman, Kalispell, Missoula), North Dakota (Fargo, Grand Forks, Minot), and Alaska (Anchorage, Wasilla). Grand Forks and Fargo were grouped into one cluster, as Fargo clinicians often treat Grand Fork patients. All clinics provided methadone and buprenorphine treatment under similar protocols, including same-day admissions, observed daily dosing, routine counseling, regular urine drug screening, and medical follow-ups within 7 days of admission and as needed thereafter. Treatment is delivered by mental health professionals (MHPs), whose education spans from high school diplomas to advanced degrees in social work and counseling."

4b-i) Report if outcomes were (self-)assessed through online questionnaires

Clearly report if outcomes were (self-)assessed through online questionnaires (as common in web-based trials) or otherwise.

	1	2	3	4	5	
subitem not at all important	○	○	○	○	◉	essential

Clear selection

Does your paper address subitem 4b-i? *

Copy and paste relevant sections from the manuscript (include quotes in quotation marks "like this" to indicate direct quotes from your manuscript), or elaborate on this item by providing additional information not in the ms, or briefly explain why the item is not applicable/relevant for your study

"The primary outcome was treatment retention. In line with federal guidelines, patients who leave care can be readmitted within 30 days without restarting the intake process14. Therefore, a patient was considered to have dropped out—and thus not retained—if a 30-day gap in treatment began within the first 30 days after intake. The first day of that gap was considered the discontinuation date."
"Treatment continuance: A binary measure defined as receiving at least 75% of expected doses within the first 30 days following intake, and at least one dose administered during the final week of that period (i.e. days 24-30 post-intake)."
"3, 7, and 30 days doses: representing the number of confirmed daily MOUD doses administered during the first 3, 7, and 30 days after intake, respectively. Dose analyses are exclusive to patients retained for at least the matching number of days. These outcomes provide a more granular view of early medication adherence patterns. Confirmed doses included both in-clinic administrations and verified take-home doses, based on standard documentation protocols in place across clinics."

4b-ii) Report how institutional affiliations are displayed

Report how institutional affiliations are displayed to potential participants [on ehealth media], as affiliations with prestigious hospitals or universities may affect volunteer rates, use, and reactions with regards to an intervention.(Not a required item – describe only if this may bias results)

	1	2	3	4	5	
subitem not at all important	○	○	◉	○	○	essential

Clear selection

Does your paper address subitem 4b-ii?

Copy and paste relevant sections from the manuscript (include quotes in quotation marks "like this" to indicate direct quotes from your manuscript), or elaborate on this item by providing additional information not in the ms, or briefly explain why the item is not applicable/relevant for your study

N/A - participants used the Recovery Connect app as part of their standard of care at Community Medical Services.

5) The interventions for each group with sufficient details to allow replication, including how and when they were actually administered

5-i) Mention names, credential, affiliations of the developers, sponsors, and owners

Mention names, credential, affiliations of the developers, sponsors, and owners [6] (if authors/evaluators are owners or developer of the software, this needs to be declared in a "Conflict of interest" section or mentioned elsewhere in the manuscript).

	1	2	3	4	5	
subitem not at all important	○	○	○	●	○	essential

Clear selection

Does your paper address subitem 5-i?

Copy and paste relevant sections from the manuscript (include quotes in quotation marks "like this" to indicate direct quotes from your manuscript), or elaborate on this item by providing additional information not in the ms, or briefly explain why the item is not applicable/relevant for your study

"Authors JP, SL and JT are employees of Bright Therapeutics, developers of Recovery Path and Recovery Connect."

5-ii) Describe the history/development process

Describe the history/development process of the application and previous formative evaluations (e.g., focus groups, usability testing), as these will have an impact on adoption/use rates and help with interpreting results.

	1	2	3	4	5	
subitem not at all important	○	○	◉	○	○	essential

Clear selection

Does your paper address subitem 5-ii?

Copy and paste relevant sections from the manuscript (include quotes in quotation marks "like this" to indicate direct quotes from your manuscript), or elaborate on this item by providing additional information not in the ms, or briefly explain why the item is not applicable/relevant for your study

"RC (Version 1.4.3) is a white-labelled version of Recovery Path, rebranded to allow partner organizations to maintain brand continuity while leveraging its evidence-based digital infrastructure. Recovery Path is a HIPAA-compliant digital remote patient monitoring platform that supports a blended care model for MOUD by combining in-person treatment with continuous digital engagement."

5-iii) Revisions and updating

Revisions and updating. Clearly mention the date and/or version number of the application/intervention (and comparator, if applicable) evaluated, or describe whether the intervention underwent major changes during the evaluation process, or whether the development and/or content was “frozen” during the trial. Describe dynamic components such as news feeds or changing content which may have an impact on the replicability of the intervention (for unexpected events see item 3b).

	1	2	3	4	5	
subitem not at all important	○	○	○	◉	○	essential

Clear selection

Does your paper address subitem 5-iii?

Copy and paste relevant sections from the manuscript (include quotes in quotation marks "like this" to indicate direct quotes from your manuscript), or elaborate on this item by providing additional information not in the ms, or briefly explain why the item is not applicable/relevant for your study

RC (Version 1.4.3) is mentioned in the ms, and no major changes occurred during the study period.

5-iv) Quality assurance methods

Provide information on quality assurance methods to ensure accuracy and quality of information provided [1], if applicable.

	1	2	3	4	5	
subitem not at all important	○	○	○	○	◉	essential

Clear selection

Does your paper address subitem 5-iv?

Copy and paste relevant sections from the manuscript (include quotes in quotation marks "like this" to indicate direct quotes from your manuscript), or elaborate on this item by providing additional information not in the ms, or briefly explain why the item is not applicable/relevant for your study

"It undergoes continuous, strict quality assurance methods to test for bugs, performance, and overall user experience, ensuring also ongoing accuracy of clinical content"

5-v) Ensure replicability by publishing the source code, and/or providing screenshots/screen-capture video, and/or providing flowcharts of the algorithms used

Ensure replicability by publishing the source code, and/or providing screenshots/screen-capture video, and/or providing flowcharts of the algorithms used. Replicability (i.e., other researchers should in principle be able to replicate the study) is a hallmark of scientific reporting.

	1	2	3	4	5	
subitem not at all important	○	○	○	●	○	essential

Clear selection

Does your paper address subitem 5-v?

Copy and paste relevant sections from the manuscript (include quotes in quotation marks "like this" to indicate direct quotes from your manuscript), or elaborate on this item by providing additional information not in the ms, or briefly explain why the item is not applicable/relevant for your study

"A full overview of data transformations and underlying statistical considerations is available in Multimedia Appendix 1, alongside the code syntax for all analyses"

5-vi) Digital preservation

Digital preservation: Provide the URL of the application, but as the intervention is likely to change or disappear over the course of the years; also make sure the intervention is archived (Internet Archive, webcitation.org, and/or publishing the source code or screenshots/videos alongside the article). As pages behind login screens cannot be archived, consider creating demo pages which are accessible without login.

	1	2	3	4	5	
subitem not at all important	○	○	○	●	○	essential

Clear selection

Does your paper address subitem 5-vi?

Copy and paste relevant sections from the manuscript (include quotes in quotation marks "like this" to indicate direct quotes from your manuscript), or elaborate on this item by providing additional information not in the ms, or briefly explain why the item is not applicable/relevant for your study

URL of Recovery Connect: https://communitymedicalservices.org/recovery-connect-app/

5-vii) Access

Access: Describe how participants accessed the application, in what setting/context, if they had to pay (or were paid) or not, whether they had to be a member of specific group. If known, describe how participants obtained "access to the platform and Internet" [1]. To ensure access for editors/reviewers/readers, consider to provide a "backdoor" login account or demo mode for reviewers/readers to explore the application (also important for archiving purposes, see vi).

	1	2	3	4	5	
subitem not at all important	○	○	○	◉	○	essential

Clear selection

Does your paper address subitem 5-vii? *

Copy and paste relevant sections from the manuscript (include quotes in quotation marks "like this" to indicate direct quotes from your manuscript), or elaborate on this item by providing additional information not in the ms, or briefly explain why the item is not applicable/relevant for your study

"Implementation involved two components: MHP training and patient onboarding. MHPs received live virtual training on app features, linkage procedures, and app-based communication strategies. Materials included step-by-step guides, video tutorials, and ongoing technical support. The training was deliberately designed to accommodate differing levels of prior experience, ensuring every MHP gains the skills to use the app effectively in clinical settings. App rollout began immediately after training, at which point MHPs were instructed to help newly admitted patients download the RC app and link via a QR code during onboarding."
"Recovery Path patient version freely available to download on Google Play and the App Store in the United States".
We are happy to provide a demo clinician account of Recovery Connect / Recovery Path to reviewers to explore.

5-viii) Mode of delivery, features/functionalities/components of the intervention and comparator, and the theoretical framework

Describe mode of delivery, features/functionalities/components of the intervention and comparator, and the theoretical framework [6] used to design them (instructional strategy [1], behaviour change techniques, persuasive features, etc., see e.g., [7, 8] for terminology). This includes an in-depth description of the content (including where it is coming from and who developed it) [1],” whether [and how] it is tailored to individual circumstances and allows users to track their progress and receive feedback” [6]. This also includes a description of communication delivery channels and – if computer-mediated communication is a component – whether communication was synchronous or asynchronous [6]. It also includes information on presentation strategies [1], including page design principles, average amount of text on pages, presence of hyperlinks to other resources, etc. [1].

	1	2	3	4	5	
subitem not at all important	○	○	○	●	○	essential

Clear selection

Does your paper address subitem 5-viii? *

Copy and paste relevant sections from the manuscript (include quotes in quotation marks "like this" to indicate direct quotes from your manuscript), or elaborate on this item by providing additional information not in the ms, or briefly explain why the item is not applicable/relevant for your study

"It enables daily check-ins, access to evidence-based resources and coping strategies, and secure, real-time communication between patients and their care teams. MHPs gain timely insights through real-time data on patient progress, risks and setbacks, allowing for early intervention and timely containment during periods of heightened distress. Between sessions, MHPs can recommend tailored coping strategies and psychoeducational content, reinforcing accountability and enhancing continuity of care. RC was also integrated into the CMS medical record system to reduce administrative burden on MHPs and improve documentation accuracy through auto-generated case notes.

5-ix) Describe use parameters

Describe use parameters (e.g., intended "doses" and optimal timing for use). Clarify what instructions or recommendations were given to the user, e.g., regarding timing, frequency, heaviness of use, if any, or was the intervention used ad libitum.

	1	2	3	4	5	
subitem not at all important	○	○	○	◉	○	essential

Clear selection

Does your paper address subitem 5-ix?

Copy and paste relevant sections from the manuscript (include quotes in quotation marks "like this" to indicate direct quotes from your manuscript), or elaborate on this item by providing additional information not in the ms, or briefly explain why the item is not applicable/relevant for your study

"MHPs were also asked to show patients key features of RC, including daily check-ins, homework activities, and the messaging feature."
There was no specific 'minimal dose' given, since this is not known. Clinicians (MHPs) are trained to encourage use of the app but patients use it ad libitum.

5-x) Clarify the level of human involvement

Clarify the level of human involvement (care providers or health professionals, also technical assistance) in the e-intervention or as co-intervention (detail number and expertise of professionals involved, if any, as well as “type of assistance offered, the timing and frequency of the support, how it is initiated, and the medium by which the assistance is delivered”. It may be necessary to distinguish between the level of human involvement required for the trial, and the level of human involvement required for a routine application outside of a RCT setting (discuss under item 21 – generalizability).

	1	2	3	4	5	
subitem not at all important	○	○	○	◉	○	essential

Clear selection

Does your paper address subitem 5-x?

Copy and paste relevant sections from the manuscript (include quotes in quotation marks "like this" to indicate direct quotes from your manuscript), or elaborate on this item by providing additional information not in the ms, or briefly explain why the item is not applicable/relevant for your study

"Implementation involved two components: MHP training and patient onboarding. MHPs received live virtual training on app features, linkage procedures, and app-based communication strategies. Materials included step-by-step guides, video tutorials, and ongoing technical support. The training was deliberately designed to accommodate differing levels of prior experience, ensuring every MHP gains the skills to use the app effectively in clinical settings. App rollout began immediately after training, at which point MHPs were instructed to help newly admitted patients download the RC app and link via a QR code during onboarding. MHPs were also asked to show patients key features of RC, including daily check-ins, homework activities, and the messaging feature."

5-xi) Report any prompts/reminders used

Report any prompts/reminders used: Clarify if there were prompts (letters, emails, phone calls, SMS) to use the application, what triggered them, frequency etc. It may be necessary to distinguish between the level of prompts/reminders required for the trial, and the level of prompts/reminders for a routine application outside of a RCT setting (discuss under item 21 – generalizability).

	1	2	3	4	5	
subitem not at all important	○	○	○	◉	○	essential

Clear selection

Does your paper address subitem 5-xi? *

Copy and paste relevant sections from the manuscript (include quotes in quotation marks "like this" to indicate direct quotes from your manuscript), or elaborate on this item by providing additional information not in the ms, or briefly explain why the item is not applicable/relevant for your study

N/A: No prompts were used during the trial. The purpose was to have a pragmatic, real-world evaluation and the study team did not encourage use of the app at any point.

5-xii) Describe any co-interventions (incl. training/support)

Describe any co-interventions (incl. training/support): Clearly state any interventions that are provided in addition to the targeted eHealth intervention, as ehealth intervention may not be designed as stand-alone intervention. This includes training sessions and support [1]. It may be necessary to distinguish between the level of training required for the trial, and the level of training for a routine application outside of a RCT setting (discuss under item 21 – generalizability.

	1	2	3	4	5	
subitem not at all important	○	○	○	◉	○	essential

Clear selection

Does your paper address subitem 5-xii? *

Copy and paste relevant sections from the manuscript (include quotes in quotation marks "like this" to indicate direct quotes from your manuscript), or elaborate on this item by providing additional information not in the ms, or briefly explain why the item is not applicable/relevant for your study

N/A: No co-interventions were involved.

6a) Completely defined pre-specified primary and secondary outcome measures, including how and when they were assessed

Does your paper address CONSORT subitem 6a? *

Copy and paste relevant sections from the manuscript (include quotes in quotation marks "like this" to indicate direct quotes from your manuscript), or elaborate on this item by providing additional information not in the ms, or briefly explain why the item is not applicable/relevant for your study

"Primary Outcome
The primary outcome was treatment retention. In line with federal guidelines, patients who leave care can be readmitted within 30 days without restarting the intake process14. Therefore, a patient was considered to have dropped out—and thus not retained—if a 30-day gap in treatment began within the first 30 days after intake. The first day of that gap was considered the discontinuation date.
Secondary Outcomes
Treatment continuance: A binary measure defined as receiving at least 75% of expected doses within the first 30 days following intake, and at least one dose administered during the final week of that period (i.e. days 24-30 post-intake).
3, 7, and 30 days doses: representing the number of confirmed daily MOUD doses administered during the first 3, 7, and 30 days after intake, respectively. Dose analyses are exclusive to patients retained for at least the matching number of days. These outcomes provide a more granular view of early medication adherence patterns. Confirmed doses included both in-clinic administrations and verified take-home doses, based on standard documentation protocols in place across clinics."

6a-i) Online questionnaires: describe if they were validated for online use and apply CHERRIES items to describe how the questionnaires were designed/deployed

If outcomes were obtained through online questionnaires, describe if they were validated for online use and apply CHERRIES items to describe how the questionnaires were designed/deployed [9].

	1	2	3	4	5	
subitem not at all important	○	○	◉	○	○	essential

Clear selection

Does your paper address subitem 6a-i?

Copy and paste relevant sections from manuscript text

N/A: Outcomes were not obtained via online questionnaires

6a-ii) Describe whether and how "use" (including intensity of use/dosage) was defined/measured/monitored

Describe whether and how "use" (including intensity of use/dosage) was defined/measured/monitored (logins, logfile analysis, etc.). Use/adoption metrics are important process outcomes that should be reported in any ehealth trial.

	1	2	3	4	5	
subitem not at all important	○	○	○	◉	○	essential

Clear selection

Does your paper address subitem 6a-ii?

Copy and paste relevant sections from manuscript text

"Engagement: Defined according to app usage patterns during the first week post-linkage. This included use of meaningful components of the app (not just opening the app) such as self-monitoring entries, and secure messages sent and received by patients through the app."

6a-iii) Describe whether, how, and when qualitative feedback from participants was obtained

Describe whether, how, and when qualitative feedback from participants was obtained (e.g., through emails, feedback forms, interviews, focus groups).

	1	2	3	4	5	
subitem not at all important	○	○	○	●	○	essential

Clear selection

Does your paper address subitem 6a-iii?

Copy and paste relevant sections from manuscript text

N/A: Qualitative feedback not included in this study

6b) Any changes to trial outcomes after the trial commenced, with reasons

Does your paper address CONSORT subitem 6b? *

Copy and paste relevant sections from the manuscript (include quotes in quotation marks "like this" to indicate direct quotes from your manuscript), or elaborate on this item by providing additional information not in the ms, or briefly explain why the item is not applicable/relevant for your study

N/A: no changes were made to outcomes after commencement of trial

7a) How sample size was determined

NPT: When applicable, details of whether and how the clustering by care provides or centers was addressed

7a-i) Describe whether and how expected attrition was taken into account when calculating the sample size

Describe whether and how expected attrition was taken into account when calculating the sample size.

	1	2	3	4	5	
subitem not at all important	○	○	○	●	○	essential

Clear selection

Does your paper address subitem 7a-i?

Copy and paste relevant sections from manuscript title (include quotes in quotation marks "like this" to indicate direct quotes from your manuscript), or elaborate on this item by providing additional information not in the ms, or briefly explain why the item is not applicable/relevant for your study

Yes, the manuscript addresses how sample size was determined and how attrition was taken into account. Specifically, the manuscript clarifies via diagrammatic representation (Figure 1) how the sample size was determined given eligibility criteria and intervention assignment. Our analytical approach is described to maximize inferential value given missing data by handling censoring at the daily level for each participant.
“We used intent-to-treat analyses, in which all enrolled patients whose sites engaged in training activities were included unless previously excluded based on exclusion criteria. The effects of exposure and linkage on odds of retention were tested using cluster-controlled discrete-time survival analyses, controlling for the nested nature of patients across sites. In these analyses, the likelihood of successful client retention on a given day, provided loss to follow-up did not occur earlier, were regressed on prior-day exposure or linkage status, with loss to follow-up defined as the event (1), retention defined as the absence of the event (0), and days after loss to follow-up defined as missing data. “

7b) When applicable, explanation of any interim analyses and stopping guidelines

Does your paper address CONSORT subitem 7b? *

Copy and paste relevant sections from the manuscript (include quotes in quotation marks "like this" to indicate direct quotes from your manuscript), or elaborate on this item by providing additional information not in the ms, or briefly explain why the item is not applicable/relevant for your study

No interim analysis of primary study outcomes, or implementation of stopping guidelines, occurred during the course of data collection.

8a) Method used to generate the random allocation sequence

NPT: When applicable, how care providers were allocated to each trial group

Does your paper address CONSORT subitem 8a? *

Copy and paste relevant sections from the manuscript (include quotes in quotation marks "like this" to indicate direct quotes from your manuscript), or elaborate on this item by providing additional information not in the ms, or briefly explain why the item is not applicable/relevant for your study

"Clusters were randomized to crossover into the intervention condition at weekly intervals beginning February 26, 2024, through April 16, 2024. We used simple randomization without replacement, generating a random permutation of all clusters in Microsoft Excel (RAND function), sorted clusters by the random number, and assigned them sequentially to the intervention condition."

8b) Type of randomisation; details of any restriction (such as blocking and block size)

Does your paper address CONSORT subitem 8b? *

Copy and paste relevant sections from the manuscript (include quotes in quotation marks "like this" to indicate direct quotes from your manuscript), or elaborate on this item by providing additional information not in the ms, or briefly explain why the item is not applicable/relevant for your study

"Clusters were randomized to crossover into the intervention condition at weekly intervals beginning February 26, 2024, through April 16, 2024. We used simple randomization without replacement, generating a random permutation of all clusters in Microsoft Excel (RAND function), sorted clusters by the random number, and assigned them sequentially to the intervention condition. The randomization algorithm and allocation schedule were finalized and archived before enrolment and implementation."

9) Mechanism used to implement the random allocation sequence (such as sequentially numbered containers), describing any steps taken to conceal the sequence until interventions were assigned

Does your paper address CONSORT subitem 9? *

Copy and paste relevant sections from the manuscript (include quotes in quotation marks "like this" to indicate direct quotes from your manuscript), or elaborate on this item by providing additional information not in the ms, or briefly explain why the item is not applicable/relevant for your study

"To preserve allocation concealment, the random sequence was created by an investigator (RS) not involved in recruitment or delivery, and the crossover dates were released to sites only as required for scheduling."

10) Who generated the random allocation sequence, who enrolled participants, and who assigned participants to interventions

Does your paper address CONSORT subitem 10? *

Copy and paste relevant sections from the manuscript (include quotes in quotation marks "like this" to indicate direct quotes from your manuscript), or elaborate on this item by providing additional information not in the ms, or briefly explain why the item is not applicable/relevant for your study

"To preserve allocation concealment, the random sequence was created by an investigator (RS) not involved in recruitment or delivery, and the crossover dates were released to sites only as required for scheduling."
"The stepped-wedge approach was selected to facilitate pragmatic implementation, reflecting real-world conditions, whilst preserving the ability to evaluate causal effects. This design also allowed us to account for temporal trends and clinic-level variability through staggered rollout."

11a) If done, who was blinded after assignment to interventions (for example, participants, care providers, those assessing outcomes) and how

NPT: Whether or not administering co-interventions were blinded to group assignment

11a-i) Specify who was blinded, and who wasn't

Specify who was blinded, and who wasn't. Usually, in web-based trials it is not possible to blind the participants [1, 3] (this should be clearly acknowledged), but it may be possible to blind outcome assessors, those doing data analysis or those administering co-interventions (if any).

	1	2	3	4	5	
subitem not at all important	○	○	○	◉	○	essential

Clear selection

Does your paper address subitem 11a-i? *

Copy and paste relevant sections from the manuscript (include quotes in quotation marks "like this" to indicate direct quotes from your manuscript), or elaborate on this item by providing additional information not in the ms, or briefly explain why the item is not applicable/relevant for your study

"Because the intervention consisted of implementing a digital tool into routine care workflows, blinding of care providers and patients was not feasible"

11a-ii) Discuss e.g., whether participants knew which intervention was the "intervention of interest" and which one was the "comparator"

Informed consent procedures (4a-ii) can create biases and certain expectations - discuss e.g., whether participants knew which intervention was the "intervention of interest" and which one was the "comparator".

	1	2	3	4	5	
subitem not at all important	○	○	○	◉	○	essential

Clear selection

Does your paper address subitem 11a-ii?

Copy and paste relevant sections from the manuscript (include quotes in quotation marks "like this" to indicate direct quotes from your manuscript), or elaborate on this item by providing additional information not in the ms, or briefly explain why the item is not applicable/relevant for your study

"The implementation of the Recovery Connect app was part of the rollout at participating clinics and integrated into existing treatment pathways. No compensation was given to clinicians as this was part of their regular duties as members of the clinics and the research did not ask for anything more than what they would otherwise do as normal practice within the clinic."

11b) If relevant, description of the similarity of interventions

(this item is usually not relevant for ehealth trials as it refers to similarity of a placebo or sham intervention to a active medication/intervention)

Does your paper address CONSORT subitem 11b? *

Copy and paste relevant sections from the manuscript (include quotes in quotation marks "like this" to indicate direct quotes from your manuscript), or elaborate on this item by providing additional information not in the ms, or briefly explain why the item is not applicable/relevant for your study

Not relevant for this study.

12a) Statistical methods used to compare groups for primary and secondary outcomes

NPT: When applicable, details of whether and how the clustering by care providers or centers was addressed

Does your paper address CONSORT subitem 12a? *

Copy and paste relevant sections from the manuscript (include quotes in quotation marks "like this" to indicate direct quotes from your manuscript), or elaborate on this item by providing additional information not in the ms, or briefly explain why the item is not applicable/relevant for your study

For the primary outcome (effect of intervention compared to control), due to the nested nature of the data and due to censoring of patient data over the course of retention, we used cluster-controlled discrete-time survival modeling to model the probability of retention on a given client-day based on exposure to the intervention on the prior day, in analyses that controlled for clustering of patients and providers within partner organizations.
“We used intent-to-treat analyses, in which all enrolled patients whose sites engaged in training activities were included unless previously excluded based on exclusion criteria. The effects of exposure and linkage on odds of retention were tested using cluster-controlled discrete-time survival analyses, controlling for the nested nature of patients across sites. In these analyses, the likelihood of successful client retention on a given day, provided loss to follow-up did not occur earlier, were regressed on prior-day exposure or linkage status, with loss to follow-up defined as the event (1), retention defined as the absence of the event (0), and days after loss to follow-up defined as missing data. … All MHP- and patient-level analyses were conducted in Mplus 8.9 using full-information maximum likelihood (FIML) estimation with a robust sandwich estimator to allow non-parametric adjustment for clustering by site, using a design-based adjustment to estimation of standard errors for nesting and for potential violations of multivariate normality. FIML allows for estimation under the assumption of Missing at Random, meaning that valid inferences can be drawn in the presence of missing data on outcomes, if known predictors of missing data are included in the model."
Regarding secondary outcomes, we also used cluster-controlled discrete-time survival modeling to evaluate the association between prior linkage and retention, and we used cluster-controlled logistic and linear regression to evaluate the association between engagement and retention, as well as the effect of exposure on secondary outcomes (treatment continuance as well as MOUD doses at 3, 7, and 30 days).
“The effects of app engagement on retention, as well as the effects of provider intervention exposure and patient linkage on secondary outcomes were assessed using cluster-controlled logistic and linear regression analyses”

12a-i) Imputation techniques to deal with attrition / missing values

Imputation techniques to deal with attrition / missing values: Not all participants will use the intervention/comparator as intended and attrition is typically high in ehealth trials. Specify how participants who did not use the application or dropped out from the trial were treated in the statistical analysis (a complete case analysis is strongly discouraged, and simple imputation techniques such as LOCF may also be problematic [4]).

	1	2	3	4	5	
subitem not at all important	○	○	○	◉	○	essential

Clear selection

Does your paper address subitem 12a-i? *

Copy and paste relevant sections from the manuscript (include quotes in quotation marks "like this" to indicate direct quotes from your manuscript), or elaborate on this item by providing additional information not in the ms, or briefly explain why the item is not applicable/relevant for your study

Yes, our manuscript addresses techniques to accommodate missing data. No multiple imputation approach was used in this manuscript;
“ll MHP- and patient-level analyses were conducted in Mplus 8.9 using full-information maximum likelihood (FIML) estimation with a robust sandwich estimator to allow non-parametric adjustment for clustering by site, using a design-based adjustment to estimation of standard errors for nesting and for potential violations of multivariate normality. FIML allows for estimation under the assumption of Missing at Random, meaning that valid inferences can be drawn in the presence of missing data on outcomes, if known predictors of missing data are included in the model.”
Our daily-level discrete-time survival analysis ensures that participants that did not use the application or dropped out contributed all appropriate data to the analysis in determining the intervention effect.

12b) Methods for additional analyses, such as subgroup analyses and adjusted analyses

Does your paper address CONSORT subitem 12b? *

Copy and paste relevant sections from the manuscript (include quotes in quotation marks "like this" to indicate direct quotes from your manuscript), or elaborate on this item by providing additional information not in the ms, or briefly explain why the item is not applicable/relevant for your study

Yes, our intervention addresses methods for moderator testing and adjusted models. First, our analytical approach used a moderator-first approach to ensure that exposure effects accurately reflected intervention effects on all participating units regardless of individual-level differences:
“Main effects analyses were tested across a series of models that estimated the effect of condition unadjusted and adjusted for covariates, following a moderator-first approach to ensure treatment effects were consistent across strata of predictors. The effects of exposure, linkage, and engagement were first modeled as a function of its main effect, as well as its interaction with each covariate in separate models. We did not hypothesize specific interaction effects; therefore, only interactions that were significant at Benjamini-Hochberg-adjusted p-values were considered non-spurious, and retained in the final adjusted model that featured all covariates.“
Further, a set of appropriate covariates were chosen to be included in adjusted models to address potential skew in conditional assignment:
“All covariates were assessed at time of patient admission, and included age, gender, ethnicity, presence of any fentanyl or amphetamine use, presence of moderate-to-severe depression or anxiety, unemployment or unhoused status, having any prior CMS admissions, number of prior CMS admissions, and primary MOUD medication (methadone or buprenorphine).”

X26) REB/IRB Approval and Ethical Considerations [recommended as subheading under "Methods"] (not a CONSORT item)

X26-i) Comment on ethics committee approval

	1	2	3	4	5	
subitem not at all important	○	○	○	○	◉	essential

Clear selection

Does your paper address subitem X26-i?

Copy and paste relevant sections from the manuscript (include quotes in quotation marks "like this" to indicate direct quotes from your manuscript), or elaborate on this item by providing additional information not in the ms, or briefly explain why the item is not applicable/relevant for your study

"The trial protocol was approved by Sterling Institutional Review Board (IRB) ID: 11629"

x26-ii) Outline informed consent procedures

Outline informed consent procedures e.g., if consent was obtained offline or online (how? Checkbox, etc.?), and what information was provided (see 4a-ii). See [6] for some items to be included in informed consent documents.

	1	2	3	4	5	
subitem not at all important	○	○	○	◉	○	essential

Clear selection

Does your paper address subitem X26-ii?

Copy and paste relevant sections from the manuscript (include quotes in quotation marks "like this" to indicate direct quotes from your manuscript), or elaborate on this item by providing additional information not in the ms, or briefly explain why the item is not applicable/relevant for your study

"Clinicians signed informed consent online to participate in the study. Patients give consent for their anonymized data to be used for research purposes as part of their treatment contract and thus a waiver for consent was approved by the IRB"

X26-iii) Safety and security procedures

Safety and security procedures, incl. privacy considerations, and any steps taken to reduce the likelihood or detection of harm (e.g., education and training, availability of a hotline)

	1	2	3	4	5	
subitem not at all important	○	○	○	●	○	essential

Clear selection

Does your paper address subitem X26-iii?

Copy and paste relevant sections from the manuscript (include quotes in quotation marks "like this" to indicate direct quotes from your manuscript), or elaborate on this item by providing additional information not in the ms, or briefly explain why the item is not applicable/relevant for your study

"All collected data, including app usage statistics and clinical outcomes, is stored securely in compliance with applicable data protection regulations. Digital data is encrypted and stored on secure servers with restricted access"
"Recovery Path (patient version freely available to download on Google Play and the App Store in the United States) is a HIPAA-compliant digital remote patient monitoring platform that supports a blended care model for MOUD by combining in-person treatment with continuous digital engagement. It undergoes continuous, strict quality assurance methods to test for bugs, performance, and overall user experience, ensuring also ongoing accuracy of clinical content."

RESULTS

13a) For each group, the numbers of participants who were randomly assigned, received intended treatment, and were analysed for the primary outcome

NPT: The number of care providers or centers performing the intervention in each group and the number of patients treated by each care provider in each center

Does your paper address CONSORT subitem 13a? *

Copy and paste relevant sections from the manuscript (include quotes in quotation marks "like this" to indicate direct quotes from your manuscript), or elaborate on this item by providing additional information not in the ms, or briefly explain why the item is not applicable/relevant for your study

Yes, Figure 2 includes a diagrammatic representation of the numbers of participants randomly assigned to intervention as opposed to control, and who were analyzed for the primary outcome.

13b) For each group, losses and exclusions after randomisation, together with reasons

Does your paper address CONSORT subitem 13b? (NOTE: Preferably, this is shown in a CONSORT flow diagram) *

Copy and paste relevant sections from the manuscript (include quotes in quotation marks "like this" to indicate direct quotes from your manuscript), or elaborate on this item by providing additional information not in the ms, or briefly explain why the item is not applicable/relevant for your study

Yes, our manuscript addresses losses and exclusions following randomization. No exclusions of participants occurred following randomization. Due to the nature of analysis, loss to follow-up was the primary outcome evaluated to determine the intervention effect.
“The primary outcome was treatment retention. In line with federal guidelines, patients who leave care can be readmitted within 30 days without restarting the intake process14. Therefore, a patient was considered to have dropped out—and thus not retained—if a 30-day gap in treatment began within the first 30 days after intake. The first day of that gap was considered the discontinuation date.”
Following our analytical approach, we prospectively predicted the occurrence of the discontinuation date using the prior day’s exposure status.

13b-i) Attrition diagram

Strongly recommended: An attrition diagram (e.g., proportion of participants still logging in or using the intervention/comparator in each group plotted over time, similar to a survival curve) or other figures or tables demonstrating usage/dose/engagement.

	1	2	3	4	5	
subitem not at all important	○	○	◉	○	○	essential

Clear selection

Does your paper address subitem 13b-i?

Copy and paste relevant sections from the manuscript or cite the figure number if applicable (include quotes in quotation marks "like this" to indicate direct quotes from your manuscript), or elaborate on this item by providing additional information not in the ms, or briefly explain why the item is not applicable/relevant for your study

While levels of engagement are operationalized as a predictor, and while treatment continuation is investigated as a secondary outcome, the study does not feature investigation of use of the intervention as an outcome measure based on exposure to the intervention, as that was outside the scope of the current manuscript and outside of the scope of analytical activities proposed in the Clinical Trial registration.

14a) Dates defining the periods of recruitment and follow-up

Does your paper address CONSORT subitem 14a? *

Copy and paste relevant sections from the manuscript (include quotes in quotation marks "like this" to indicate direct quotes from your manuscript), or elaborate on this item by providing additional information not in the ms, or briefly explain why the item is not applicable/relevant for your study

Yes, our manuscript addresses dates regarding recruitment and follow-up. Figure 1 of the manuscript details the study time period, cross-over points for participating organizations, and the manuscript addresses how 30-day retention following enrollment during the study time period was assessed.

14a-i) Indicate if critical “secular events” fell into the study period

Indicate if critical “secular events” fell into the study period, e.g., significant changes in Internet resources available or “changes in computer hardware or Internet delivery resources”

	1	2	3	4	5	
subitem not at all important	○	○	○	●	○	essential

Clear selection

Does your paper address subitem 14a-i?

Copy and paste relevant sections from the manuscript (include quotes in quotation marks "like this" to indicate direct quotes from your manuscript), or elaborate on this item by providing additional information not in the ms, or briefly explain why the item is not applicable/relevant for your study

No critical secular events occurred during the study period.

14b) Why the trial ended or was stopped (early)

Does your paper address CONSORT subitem 14b? *

Copy and paste relevant sections from the manuscript (include quotes in quotation marks "like this" to indicate direct quotes from your manuscript), or elaborate on this item by providing additional information not in the ms, or briefly explain why the item is not applicable/relevant for your study

Our manuscript defines the end of the study period in which data collection occurred (see Figure 1). The length of the study period was chosen as sufficient for robust inference regarding intervention effect on 30-day retention, based on the number of participants included in control and intervention groups.

15) A table showing baseline demographic and clinical characteristics for each group

NPT: When applicable, a description of care providers (case volume, qualification, expertise, etc.) and centers (volume) in each group

Does your paper address CONSORT subitem 15? *

Copy and paste relevant sections from the manuscript (include quotes in quotation marks "like this" to indicate direct quotes from your manuscript), or elaborate on this item by providing additional information not in the ms, or briefly explain why the item is not applicable/relevant for your study

Yes; Table 1 shows baseline demographic and clinical characteristics for the patient group.

15-i) Report demographics associated with digital divide issues

In ehealth trials it is particularly important to report demographics associated with digital divide issues, such as age, education, gender, social-economic status, computer/Internet/ehealth literacy of the participants, if known.

	1	2	3	4	5	
subitem not at all important	○	○	◉	○	○	essential

Clear selection

Does your paper address subitem 15-i? *

Copy and paste relevant sections from the manuscript (include quotes in quotation marks "like this" to indicate direct quotes from your manuscript), or elaborate on this item by providing additional information not in the ms, or briefly explain why the item is not applicable/relevant for your study

We report age, race and gender, as well as socio-economic metrics such as housing and employment. Computer literacy was not known and therefore not reported.

16) For each group, number of participants (denominator) included in each analysis and whether the analysis was by original assigned groups

16-i) Report multiple “denominators” and provide definitions

Report multiple “denominators” and provide definitions: Report N’s (and effect sizes) “across a range of study participation [and use] thresholds” [1], e.g., N exposed, N consented, N used more than x times, N used more than y weeks, N participants “used” the intervention/comparator at specific pre-defined time points of interest (in absolute and relative numbers per group). Always clearly define “use” of the intervention.

	1	2	3	4	5	
subitem not at all important	○	○	○	◉	○	essential

Clear selection

Does your paper address subitem 16-i? *

Copy and paste relevant sections from the manuscript (include quotes in quotation marks "like this" to indicate direct quotes from your manuscript), or elaborate on this item by providing additional information not in the ms, or briefly explain why the item is not applicable/relevant for your study

Yes, the manuscript refers to all denominators (sample sizes) appropriate to various analyses; the manuscript indicates that the full sample (n=1524) was used for primary outcome analyses, and appropriate subsets were used for specific secondary analyses. Tables 3 and 4 , as well as supplemental tables, explicitly mention all denominators for given analyses reported in those tables.

16-ii) Primary analysis should be intent-to-treat

Primary analysis should be intent-to-treat, secondary analyses could include comparing only “users”, with the appropriate caveats that this is no longer a randomized sample (see 18-i).

	1	2	3	4	5	
subitem not at all important	○	○	○	○	○	essential

Does your paper address subitem 16-ii?

Copy and paste relevant sections from the manuscript (include quotes in quotation marks "like this" to indicate direct quotes from your manuscript), or elaborate on this item by providing additional information not in the ms, or briefly explain why the item is not applicable/relevant for your study

“We used intent-to-treat analyses, in which all enrolled patients whose sites engaged in training activities were included unless previously excluded based on exclusion criteria. The effects of exposure and linkage on odds of retention were tested using cluster-controlled discrete-time survival analyses, controlling for the nested nature of patients across sites. In these analyses, the likelihood of successful client retention on a given day, provided loss to follow-up did not occur earlier, were regressed on prior-day exposure or linkage status, with loss to follow-up defined as the event (1), retention defined as the absence of the event (0), and days after loss to follow-up defined as missing data. The effects of app engagement on retention, as well as the effects of provider intervention exposure and patient linkage on secondary outcomes were assessed using cluster-controlled logistic and linear regression analyses. Effects of exposure were examined across all patients based on their degree of exposure. Effects of linkage and engagement were examined across all patients, as well as among those fully exposed. This allowed us to study both the pragmatic, system-level impact of linkage and engagement under routine conditions, including partial exposure and implementation variability, as well as analyze the fully exposed subgroup to isolate effects under implementation fidelity.“

17a) For each primary and secondary outcome, results for each group, and the estimated effect size and its precision (such as 95% confidence interval)

Does your paper address CONSORT subitem 17a? *

Copy and paste relevant sections from the manuscript (include quotes in quotation marks "like this" to indicate direct quotes from your manuscript), or elaborate on this item by providing additional information not in the ms, or briefly explain why the item is not applicable/relevant for your study

Yes; Tables 3 and 4, as well as supplemental tables, report effect sizes and confidence intervals of all regression effects on all study primary and secondary outcomes.

17a-i) Presentation of process outcomes such as metrics of use and intensity of use

In addition to primary/secondary (clinical) outcomes, the presentation of process outcomes such as metrics of use and intensity of use (dose, exposure) and their operational definitions is critical. This does not only refer to metrics of attrition (13-b) (often a binary variable), but also to more continuous exposure metrics such as "average session length". These must be accompanied by a technical description how a metric like a "session" is defined (e.g., timeout after idle time) [1] (report under item 6a).

	1	2	3	4	5	
subitem not at all important	○	○	○	●	○	essential

Clear selection

Does your paper address subitem 17a-i?

Copy and paste relevant sections from the manuscript (include quotes in quotation marks "like this" to indicate direct quotes from your manuscript), or elaborate on this item by providing additional information not in the ms, or briefly explain why the item is not applicable/relevant for your study

Yes; the links between patient linkage with the app and app engagement, and treatment primary and secondary outcomes, are investigated and reported in Tables 3, 4, and Supplemental tables. In addition, the Results section states:

"Linkage
Results of linkage analyses are shown in Table 3 and Figure 4a-b.
Across full sample (N = 1524). There were no significant interactions between linkage and covariates (ps .09 - .91). In unadjusted analyses, compared to no linkage, patients who linked were more likely to be subsequently retained at 30 days (HR (loss) = 0.63, 95% CI= 0.48 - 0.83, p<.001; 81.3% 30-day retention among those who linked compared to 72.0% retention among those unlinked). Adjusted effects of linkage were of equal valence and were significant (HR: 0.65, 95% CI: 0.48- 0.87, p=.004).
Among Exposed (n = 1209). There were no significant interactions between linkage and covariates (ps .09 - .99) after correction. In unadjusted analyses, compared to no linkage, patients who linked were more likely to be subsequently retained at 30 days (HR (loss) = 0.69, 95% CI= 0.50 - 0.95, p=.021; 80.9% 30-day retention compared to 73.5% retention among unlinked). Adjusted effects of linkage were of equal valence and significant (HR: 0.70, 95% CI: 0.51- 0.97, p=.032).

Engagement
Results of engagement analyses are shown in Table 4. There were no significant interactions between linkage and covariates (ps .04 - .89) after correction. Among those retained at 1 week (n=1366), minimal, moderate, and high engagement during that first week were each linked to greater 30-day retention compared to no engagement (unadjusted ORs=2.79-7.15, ps<.001; adjusted ORs=2.75-6.35, ps=.003 - <001). Further, unadjusted high engagement was linked to greater 30-day retention compared to minimal engagement (b = -0.941, p = .04, adjusted: b = -0.844, p = .069). Among those exposed and retained at 1 week (n = 977), minimal, moderate, and high engagement were each linked to greater retention compared to no engagement (unadjusted ORs= 2.39-6.44, ps<.001)."

17b) For binary outcomes, presentation of both absolute and relative effect sizes is recommended

Does your paper address CONSORT subitem 17b? *

Copy and paste relevant sections from the manuscript (include quotes in quotation marks "like this" to indicate direct quotes from your manuscript), or elaborate on this item by providing additional information not in the ms, or briefly explain why the item is not applicable/relevant for your study

Yes; Relative effect sizes are reported in text and tables 3, 4, and supplemental tables for all primary and secondary outcomes. Absolute differences in study primary and secondary outcomes are reported in Table 2; the estimated absolute difference in 30-day retention due to exposure is reported in the note of Figure 3; the estimated absolute difference in 30-day retention due to linkage is shown in Figure 4a-b.

18) Results of any other analyses performed, including subgroup analyses and adjusted analyses, distinguishing pre-specified from exploratory

Does your paper address CONSORT subitem 18? *

Copy and paste relevant sections from the manuscript (include quotes in quotation marks "like this" to indicate direct quotes from your manuscript), or elaborate on this item by providing additional information not in the ms, or briefly explain why the item is not applicable/relevant for your study

Yes; Study primary and secondary outcomes follow defined measures from the Clinical Trial registration. No additional exploratory outcomes were investigated.

18-i) Subgroup analysis of comparing only users

A subgroup analysis of comparing only users is not uncommon in ehealth trials, but if done, it must be stressed that this is a self-selected sample and no longer an unbiased sample from a randomized trial (see 16-iii).

	1	2	3	4	5	
subitem not at all important	○	○	○	●	○	essential

Clear selection

Does your paper address subitem 18-i?

Copy and paste relevant sections from the manuscript (include quotes in quotation marks "like this" to indicate direct quotes from your manuscript), or elaborate on this item by providing additional information not in the ms, or briefly explain why the item is not applicable/relevant for your study

Yes; our manuscript discusses the links between app linkage and engagement, in terms indicating that such behavior is self-selected and thus cannot be used to determine causal effects between that behavior and study outcomes.
E.g. for engagement: “Further, unadjusted high engagement was linked to greater 30-day retention compared to minimal engagement (b = -0.941, p = .04, adjusted: b = -0.844, p = .069). Among those exposed and retained at 1 week (n = 977), minimal, moderate, and high engagement were each linked to greater retention compared to no engagement (unadjusted ORs= 2.39-6.44, ps<.001).

19) All important harms or unintended effects in each group
(for specific guidance see CONSORT for harms)

Does your paper address CONSORT subitem 19? *

Copy and paste relevant sections from the manuscript (include quotes in quotation marks "like this" to indicate direct quotes from your manuscript), or elaborate on this item by providing additional information not in the ms, or briefly explain why the item is not applicable/relevant for your study

There were no important harms or unintended effects during the study.

19-i) Include privacy breaches, technical problems

Include privacy breaches, technical problems. This does not only include physical “harm” to participants, but also incidents such as perceived or real privacy breaches [1], technical problems, and other unexpected/unintended incidents. “Unintended effects” also includes unintended positive effects [2].

	1	2	3	4	5	
subitem not at all important	○	○	○	●	○	essential

Clear selection

Does your paper address subitem 19-i?

Copy and paste relevant sections from the manuscript (include quotes in quotation marks "like this" to indicate direct quotes from your manuscript), or elaborate on this item by providing additional information not in the ms, or briefly explain why the item is not applicable/relevant for your study

There were no privacy breaches or technical problems with the app during the study, nor any unintended effects.

19-ii) Include qualitative feedback from participants or observations from staff/researchers

Include qualitative feedback from participants or observations from staff/researchers, if available, on strengths and shortcomings of the application, especially if they point to unintended/unexpected effects or uses. This includes (if available) reasons for why people did or did not use the application as intended by the developers.

	1	2	3	4	5	
subitem not at all important	○	○	○	●	○	essential

Clear selection

Does your paper address subitem 19-ii?

Copy and paste relevant sections from the manuscript (include quotes in quotation marks "like this" to indicate direct quotes from your manuscript), or elaborate on this item by providing additional information not in the ms, or briefly explain why the item is not applicable/relevant for your study

For this study, there was no unintended effects of the app, and the app was used as intended as part of a natural implementation and integration into clinical workflows.
For qualitative analysis for the app and feedback from users, this has been conducted elsewhere as part of a feasibility paper currently in press (Digitally-enhanced medication-assisted treatment for opioid use disorder: Acceptability, engagement and treatment retention, Palacios et al, Frontiers in Psychiatry).

DISCUSSION

22) Interpretation consistent with results, balancing benefits and harms, and considering other relevant evidence

NPT: In addition, take into account the choice of the comparator, lack of or partial blinding, and unequal expertise of care providers or centers in each group

22-i) Restate study questions and summarize the answers suggested by the data, starting with primary outcomes and process outcomes (use)

Restate study questions and summarize the answers suggested by the data, starting with primary outcomes and process outcomes (use).

	1	2	3	4	5	
subitem not at all important	○	○	○	○	◉	essential

Clear selection

Does your paper address subitem 22-i? *

Copy and paste relevant sections from the manuscript (include quotes in quotation marks "like this" to indicate direct quotes from your manuscript), or elaborate on this item by providing additional information not in the ms, or briefly explain why the item is not applicable/relevant for your study

"The improvements in 30-day retention and treatment continuance among exposed patients indicate that implementation of RC (Recovery Path) can meaningfully enhance standard MOUD care. Based on adjusted model estimates, MHP training provided a 23% (HR of covariate-adjusted non-survival = 0.81) decrease in treatment discontinuation."
"Further, among patients whose clinicians were trained, those who linked early on via RC showed even higher retention, showing that downloading the app and linking with their MHP is associated with greater benefit beyond exposure alone. In addition, minimal to high engagement during the first week emerged as a strong and consistent predictor of positive outcomes, underscoring the critical importance of early and active engagement."

22-ii) Highlight unanswered new questions, suggest future research

Highlight unanswered new questions, suggest future research.

	1	2	3	4	5	
subitem not at all important	○	○	○	◉	○	essential

Clear selection

Does your paper address subitem 22-ii?

Copy and paste relevant sections from the manuscript (include quotes in quotation marks "like this" to indicate direct quotes from your manuscript), or elaborate on this item by providing additional information not in the ms, or briefly explain why the item is not applicable/relevant for your study

"Future studies should also explore cost-effectiveness, equity of access, and patient-centered outcomes to further define this impact."
"Future research should extend this work to examine long-term outcomes, differential effectiveness across populations, and cost effectiveness of implementing digital care models in routine practice."

20) Trial limitations, addressing sources of potential bias, imprecision, and, if relevant, multiplicity of analyses

20-i) Typical limitations in ehealth trials

Typical limitations in ehealth trials: Participants in ehealth trials are rarely blinded. Ehealth trials often look at a multiplicity of outcomes, increasing risk for a Type I error. Discuss biases due to non-use of the intervention/usability issues, biases through informed consent procedures, unexpected events.

	1	2	3	4	5	
subitem not at all important	○	○	○	●	○	essential

Clear selection

Does your paper address subitem 20-i? *

Copy and paste relevant sections from the manuscript (include quotes in quotation marks "like this" to indicate direct quotes from your manuscript), or elaborate on this item by providing additional information not in the ms, or briefly explain why the item is not applicable/relevant for your study

"First, due to the nature of the app (facilitating interaction between MHP and patient) there was no obvious method of including an attention-matched control app to be used as control. The baseline standard of care at each site served as comparator for the effect of app training and usage on retention, linkage, and engagement, as per usual stepped-wedge crossover design25. Furthermore, for feasibility reasons, the stepped-wedge design featured site transition into the experimental condition within short time frames: each site was inducted into the experimental condition with one week difference relative to each other. This raises the concern that there were unobserved consequences or correlates of the passage of time that may have affected app adoption. However, in exploratory analyses, we found no indication of any secular trends of change over time in any of our outcome measures, following either parametric or non-parametric definitions of change. We also focused on short-term outcomes (30 days); future work will examine sustained retention, relapse, and recovery indicators over longer time horizons.

Patients were identified using a global identifier, so that if they were to transfer among any of the clinics at CMS their dosing records would be continuous. The data records do not include an indication of whether a patient newly entering treatment transferred from an outside clinic and would therefore already be established on methadone at entry. The maximum allowable first day dose (30 mg before April 2024 and 50 mg afterwards) was used to classify patients as either newly started on methadone or as transfers, since most transfer patients are at doses above these levels. Some patients may be misclassified using this technique, however, this would not be expected to create bias in this stepped-wedge study design. For new patients started on buprenorphine, there was no way to determine if they had been taking it outside of CMS prior to admission. Nevertheless, patients taking buprenorphine were accounted for statistically, and represented a minority of the total sample."

21) Generalisability (external validity, applicability) of the trial findings

NPT: External validity of the trial findings according to the intervention, comparators, patients, and care providers or centers involved in the trial

21-i) Generalizability to other populations

Generalizability to other populations: In particular, discuss generalizability to a general Internet population, outside of a RCT setting, and general patient population, including applicability of the study results for other organizations

	1	2	3	4	5	
subitem not at all important	○	○	○	●	○	essential

Clear selection

Does your paper address subitem 21-i?

Copy and paste relevant sections from the manuscript (include quotes in quotation marks "like this" to indicate direct quotes from your manuscript), or elaborate on this item by providing additional information not in the ms, or briefly explain why the item is not applicable/relevant for your study

"Importantly, to date, adoption of digital tools in OUD treatment has not reached a scale sufficient to meet the needs of many individuals requiring care. Much of the existing research has centered on feasibility or efficacy in ideal conditions, leaving a critical gap in understanding how such tools can be effectively deployed into real world MOUD treatment settings. One national survey found that only 34% of U.S. healthcare organizations reported using any category of digital health technology for OUD treatment24. This study contributes to bridging that gap, demonstrating that a blended care model, where digital tools integrate into existing clinical workflows and medical record systems, can be implemented across multiple outpatient MOUD settings with measurable improvements in patient retention"

21-ii) Discuss if there were elements in the RCT that would be different in a routine application setting

Discuss if there were elements in the RCT that would be different in a routine application setting (e.g., prompts/reminders, more human involvement, training sessions or other co-interventions) and what impact the omission of these elements could have on use, adoption, or outcomes if the intervention is applied outside of a RCT setting.

	1	2	3	4	5	
subitem not at all important	○	○	◉	○	○	essential

Clear selection

Does your paper address subitem 21-ii?

Copy and paste relevant sections from the manuscript (include quotes in quotation marks "like this" to indicate direct quotes from your manuscript), or elaborate on this item by providing additional information not in the ms, or briefly explain why the item is not applicable/relevant for your study

Our study is a pragmatic, real-world RCT and the implementation, training and use of the app reflected routine clinical applications.

OTHER INFORMATION

23) Registration number and name of trial registry

Does your paper address CONSORT subitem 23? *

Copy and paste relevant sections from the manuscript (include quotes in quotation marks "like this" to indicate direct quotes from your manuscript), or elaborate on this item by providing additional information not in the ms, or briefly explain why the item is not applicable/relevant for your study

ClinicalTrials.gov ID: NCT07140926

24) Where the full trial protocol can be accessed, if available

Does your paper address CONSORT subitem 24? *

Cite a Multimedia Appendix, other reference, or copy and paste relevant sections from the manuscript (include quotes in quotation marks "like this" to indicate direct quotes from your manuscript), or elaborate on this item by providing additional information not in the ms, or briefly explain why the item is not applicable/relevant for your study

Full trial protocol is not available

25) Sources of funding and other support (such as supply of drugs), role of funders

Does your paper address CONSORT subitem 25? *

Copy and paste relevant sections from the manuscript (include quotes in quotation marks "like this" to indicate direct quotes from your manuscript), or elaborate on this item by providing additional information not in the ms, or briefly explain why the item is not applicable/relevant for your study

"Funding for this study was provided by Bright Therapeutics."

X27) Conflicts of Interest (not a CONSORT item)

X27-i) State the relation of the study team towards the system being evaluated

In addition to the usual declaration of interests (financial or otherwise), also state the relation of the study team towards the system being evaluated, i.e., state if the authors/evaluators are distinct from or identical with the developers/sponsors of the intervention.

	1	2	3	4	5	
subitem not at all important	○	○	○	○	●	essential

Clear selection

Does your paper address subitem X27-i?

Copy and paste relevant sections from the manuscript (include quotes in quotation marks "like this" to indicate direct quotes from your manuscript), or elaborate on this item by providing additional information not in the ms, or briefly explain why the item is not applicable/relevant for your study

"Authors JP, SL and JT are employees of Bright Therapeutics, developers of Recovery Path and Recovery Connect."

About the CONSORT EHEALTH checklist

As a result of using this checklist, did you make changes in your manuscript? *

- ○ yes, major changes
- ● yes, minor changes
- ○ no

What were the most important changes you made as a result of using this checklist?

Added word to the title, some clarifying information in the methods, additional information on randomization procedures.

How much time did you spend on going through the checklist INCLUDING making changes in your manuscript *

Approximately one full working day (8 hours).

As a result of using this checklist, do you think your manuscript has improved? *

- (selected) yes
- no
- Other:

Would you like to become involved in the CONSORT EHEALTH group?

This would involve for example becoming involved in participating in a workshop and writing an "Explanation and Elaboration" document

- (selected) yes
- no
- Other:

Clear selection

Any other comments or questions on CONSORT EHEALTH

Very useful, however some questions should consider different methodologies and trial designs.

STOP - Save this form as PDF before you click submit

To generate a record that you filled in this form, we recommend to generate a PDF of this page (on a Mac, simply select "print" and then select "print as PDF") before you submit it.

When you submit your (revised) paper to JMIR, please upload the PDF as supplementary file.

Don't worry if some text in the textboxes is cut off, as we still have the complete information in our database. Thank you!

Final step: Click submit !

Click submit so we have your answers in our database!

Submit Clear form

Never submit passwords through Google Forms.

This form was created outside of your domain. - Contact form owner - Terms of Service - Privacy Policy

Does this form look suspicious? Report

Google Forms
