# Supplementary material for: Implementation of a Mobile Digital Tool Supporting Medication for Opioid Use Disorder Treatment Improves Retention: Stepped-Wedge Cluster Randomized Controlled Trial
Source: J Med Internet Res. 2025 Dec 22;27:e83346. doi: 10.2196/83346 (PMC12770919; doi:10.2196/83346)
Supplement: Multimedia Appendix 3 [file jmir_v27i1e83346_app3.docx]

### Table S1. Effects of Exposure on Secondary Treatment Outcomes

|  | Treatment Continuance | | | | 3-Day Doses | | | | 7-Day Doses | | | | 30-Day Doses | | | |
| --- | --- | --- | --- | --- | --- | --- | --- | --- | --- | --- | --- | --- | --- | --- | --- | --- |
|  | HR | 95% CI | | p | B | 95% CI | | p | B | 95% CI | | p | B | 95% CI | | p |
| Unadjusted Exposure (0= not trained, 1 = trained) | 1.66 | 1.05 | 2.65 | .032 | 0.10 | >.00 | 0.20 | .037 | 0.12 | 0.03 | 0.20 | .007 | 0.10 | 0.03 | 0.18 | .007 |
| Adjusted Exposure (0= not trained, 1 = trained) | 1.70 | 1.08 | 2.67 | .022 | 0.09 | >.00 | 0.19 | .047 | 0.11 | 0.03 | 0.19 | .010 | 0.09 | 0.69 | 3.59 | .004 |
| Fentanyl-positive on admission (0=Yes, 1=No) | 1.15 | 0.80 | 1.66 | .443 | -0.07 | -0.13 | -0.01 | .019 | -0.03 | -0.10 | 0.04 | .393 | 0.00 | -1.68 | 1.75 | .971 |
| Amphetamine-positive on admission | 0.37 | 0.28 | 0.49 | >.001 | -0.13 | -0.20 | -0.05 | .003 | -0.19 | -0.26 | -0.11 | >.001 | -0.20 | -5.33 | -2.45 | >.001 |
| Moderate-to-severe depression | 0.83 | 0.59 | 1.15 | .257 | -0.04 | -0.10 | 0.03 | .302 | -0.06 | -0.14 | 0.02 | .141 | -0.05 | -3.21 | 0.59 | .178 |
| Moderate-to-severe anxiety | 0.71 | 0.47 | 1.06 | .091 | -0.01 | -0.08 | 0.05 | .682 | 0.00 | -0.05 | 0.05 | .910 | -0.04 | -2.45 | 0.47 | .184 |
| Financial stress | 0.85 | 0.56 | 1.29 | .447 | -0.05 | -0.09 | -0.01 | .007 | -0.08 | -0.11 | -0.04 | >.001 | -0.06 | -2.96 | 0.22 | .092 |
| Gender | 0.91 | 0.76 | 1.09 | .325 | 0.00 | -0.07 | 0.07 | .948 | -0.03 | -0.06 | 0.01 | .099 | -0.01 | -0.90 | 0.62 | .727 |
| Age (1=>37years; 0=<38years) | 1.39 | 1.04 | 1.86 | .026 | 0.05 | 0.01 | 0.09 | .013 | 0.05 | -0.01 | 0.10 | .085 | 0.08 | 0.54 | 2.54 | .002 |
| Has had prior admissions (1=yes, 0=no) | 1.17 | 0.85 | 1.61 | .329 | -0.09 | -0.15 | -0.03 | .003 | -0.05 | -0.12 | 0.01 | .088 | -0.01 | -1.20 | 0.93 | .803 |
| Number of prior admissions | 0.80 | 0.68 | 0.95 | .009 | -0.02 | -0.11 | 0.06 | .582 | -0.04 | -0.13 | 0.05 | .384 | -0.10 | -1.32 | -0.06 | .032 |
| Race/Ethnicity (ref: Non-Hispanic white) |  |  |  |  |  |  |  |  |  |  |  |  |  |  |  |  |
| Black or African American | 0.33 | 0.21 | 0.53 | >.001 | -0.09 | -0.18 | <.00 | .047 | -0.08 | -0.11 | -0.04 | >.001 | -0.09 | -7.39 | -3.89 | >.001 |
| Hispanic/Latino | 0.86 | 0.60 | 1.24 | .420 | -0.03 | -0.06 | >.00 | .117 | -0.03 | -0.06 | 0.00 | .089 | -0.02 | -2.82 | 0.96 | .336 |
| Native American/Pacific Islander | 0.72 | 0.53 | 0.99 | .044 | -0.03 | -0.11 | 0.05 | .494 | -0.05 | -0.13 | 0.03 | .200 | -0.08 | -3.47 | -0.24 | .024 |
| Unknown | 0.96 | 0.73 | 1.26 | .770 | -0.02 | -0.06 | 0.02 | .288 | -0.02 | -0.07 | 0.03 | .405 | -0.01 | -1.65 | 1.28 | .805 |
| Buprenorphine as MOUD (ref: Methadone) | 0.41 | 0.31 | 0.54 | >.001 | 0.00 | -0.09 | 0.08 | .928 | 0.02 | -0.08 | 0.13 | .663 | -0.12 | -5.72 | -1.83 | >.001 |

### Table S2. Effects of Linkage on Secondary Treatment Outcomes (*n* = 1378*)

|  | Treatment Continuance | | | | | 3-Day #Doses | | | | | 7-Day #Doses | | | | | 30-Day #Doses | | | | |
| --- | --- | --- | --- | --- | --- | --- | --- | --- | --- | --- | --- | --- | --- | --- | --- | --- | --- | --- | --- | --- |
|  | b | HR | 95% CI | | *p* | b | B | 95% CI | | *p* | b | B | 95% CI | | *p* | b | B | 95% CI | | *p* |
| Linked by end of period (3,7, 30 days; 0=not linked, 1=linked) | 0.77 | 2.16 | 1.85 | 2.53 | <.001 | 0.10 | 0.05 | 0.00 | 0.11 | .049 | 0.40 | 0.08 | 0.04 | 0.13 | <.001 | 4.10 | 0.19 | 0.15 | 0.24 | <.001 |
| Adjusted Linked by end of period (3,7, 30 days; 0=not linked, 1=linked) | 0.83 | 2.30 | 1.97 | 2.67 | <.001 | 0.07 | 0.04 | -0.01 | 0.09 | .116 | 0.34 | 0.07 | 0.03 | 0.11 | .001 | 3.93 | 0.19 | 0.14 | 0.23 | <.001 |
| Fentanyl-positive on admission (0=Yes, 1=No) | 0.14 | 1.15 | 0.79 | 1.69 | .468 | -0.11 | -0.08 | -0.14 | -0.01 | .020 | -0.13 | -0.03 | -0.11 | 0.04 | .367 | 0.13 | 0.01 | -0.07 | 0.09 | .885 |
| Amphetamine-positive on admission | -1.00 | 0.37 | 0.27 | 0.49 | .000 | -0.16 | -0.13 | -0.21 | -0.05 | .003 | -0.65 | -0.19 | -0.26 | -0.11 | <.001 | -3.94 | -0.21 | -0.28 | -0.13 | <.001 |
| Moderate-to-severe depression | -0.21 | 0.81 | 0.56 | 1.18 | .277 | -0.07 | -0.04 | -0.10 | 0.03 | .284 | -0.34 | -0.06 | -0.14 | 0.02 | .127 | -1.36 | -0.05 | -0.11 | 0.02 | .154 |
| Moderate-to-severe anxiety | -0.32 | 0.73 | 0.49 | 1.08 | .119 | -0.02 | -0.01 | -0.08 | 0.06 | .799 | 0.01 | 0.00 | -0.05 | 0.05 | .949 | -0.84 | -0.03 | -0.09 | 0.02 | .234 |
| Financial stress | -0.10 | 0.91 | 0.59 | 1.40 | .661 | -0.08 | -0.05 | -0.10 | -0.01 | .010 | -0.29 | -0.07 | -0.10 | -0.05 | <.001 | -1.02 | -0.05 | -0.12 | 0.03 | .221 |
| Gender | -0.14 | 0.87 | 0.74 | 1.02 | .093 | 0.00 | 0.00 | -0.07 | 0.07 | .964 | -0.10 | -0.03 | -0.06 | 0.00 | .062 | -0.37 | -0.02 | -0.06 | 0.02 | .285 |
| Age (1=>37years; 0=<38years) | 0.40 | 1.49 | 1.12 | 1.98 | .007 | 0.06 | 0.05 | 0.01 | 0.09 | .014 | 0.17 | 0.05 | -0.01 | 0.11 | .072 | 1.83 | 0.10 | 0.04 | 0.15 | <.001 |
| Has had prior admissions (1=yes, 0=no) | 0.21 | 1.24 | 0.91 | 1.68 | .173 | -0.11 | -0.09 | -0.14 | -0.03 | .003 | -0.17 | -0.05 | -0.11 | 0.01 | .089 | 0.11 | 0.01 | -0.05 | 0.06 | .841 |
| Number of prior admissions | -0.22 | 0.80 | 0.69 | 0.95 | .008 | -0.01 | -0.03 | -0.11 | 0.06 | .525 | -0.06 | -0.05 | -0.13 | 0.04 | .294 | -0.67 | -0.10 | -0.18 | -0.01 | .029 |
| Race/Ethnicity (ref: Non-Hispanic white) |  |  |  |  |  |  |  |  |  |  |  |  |  |  |  |  |  |  |  |  |
| Black or African American | -0.93 | 0.39 | 0.27 | 0.58 | <.001 | -0.35 | -0.09 | -0.17 | 0.00 | .054 | -0.78 | -0.07 | -0.11 | -0.03 | <.001 | -4.90 | -0.08 | -0.11 | -0.05 | <.001 |
| Hispanic/Latino | -0.22 | 0.80 | 0.56 | 1.13 | .207 | -0.08 | -0.03 | -0.06 | 0.01 | .139 | -0.23 | -0.03 | -0.06 | 0.01 | .122 | -1.21 | -0.03 | -0.07 | 0.02 | .241 |
| Native American/Pacific Islander | -0.31 | 0.73 | 0.52 | 1.03 | .071 | -0.04 | -0.03 | -0.10 | 0.05 | .454 | -0.22 | -0.05 | -0.13 | 0.03 | .178 | -1.75 | -0.08 | -0.15 | 0.00 | .040 |
| Unknown | -0.02 | 0.98 | 0.74 | 1.29 | .888 | -0.03 | -0.02 | -0.05 | 0.02 | .363 | -0.08 | -0.02 | -0.06 | 0.03 | .495 | -0.08 | 0.00 | -0.06 | 0.05 | .915 |
| Buprenorphine as MOUD (ref: Methadone) | -0.96 | 0.38 | 0.27 | 0.54 | <.001 | -0.01 | -0.01 | -0.09 | 0.08 | .894 | 0.11 | 0.02 | -0.09 | 0.12 | .732 | -3.98 | -0.13 | -0.19 | -0.06 | <.001 |

Note: *Those patients whose clinician was trained in the use of RC during their ongoing admission were removed from this particular analysis.

### Table S3. Effects of Linkage on Secondary Treatment Outcomes (*n* = 1089, among Exposed)

|  | TC30 | | | | 3-Day Doses | | | | 7-Day Doses | | | | 30-Day Doses | | | |
| --- | --- | --- | --- | --- | --- | --- | --- | --- | --- | --- | --- | --- | --- | --- | --- | --- |
|  | HR | 95% CI |  | *p* | B | 95% CI |  | *p* | B | 95% CI |  | *p* | B | 95% CI |  | *p* |
| Linked by end of period (3,7, 30 days; 0=not linked, 1=linked) | 1.99 | 1.66 | 2.39 | <.001 | 0.04 | -0.01 | 0.09 | .115 | 0.07 | 0.03 | 0.11 | .002 | 0.19 | 0.16 | 0.23 | <.001 |
| Adjusted Linked by end of period (3,7, 30 days; 0=not linked, 1=linked) | 2.16 | 1.79 | 2.60 | <.001 | 0.02 | -0.02 | 0.07 | .306 | 0.06 | 0.02 | 0.09 | .008 | 0.19 | 0.15 | 0.23 | <.001 |
| Fentanyl-positive on admission (0=Yes, 1=No) | 1.15 | 0.82 | 1.62 | .425 | -0.08 | -0.13 | -0.02 | .003 | -0.03 | -0.11 | 0.05 | .471 | 0.01 | -0.07 | 0.10 | .767 |
| Amphetamine-positive on admission | 0.39 | 0.29 | 0.53 | .000 | -0.11 | -0.19 | -0.03 | .012 | -0.16 | -0.24 | -0.08 | .000 | -0.19 | -0.25 | -0.13 | .000 |
| Moderate-to-severe depression | 0.88 | 0.60 | 1.30 | .531 | -0.04 | -0.12 | 0.04 | .324 | -0.07 | -0.16 | 0.03 | .158 | -0.05 | -0.13 | 0.03 | .171 |
| Moderate-to-severe anxiety | 0.66 | 0.46 | 0.96 | .030 | -0.01 | -0.06 | 0.04 | .735 | -0.01 | -0.06 | 0.05 | .807 | -0.05 | -0.11 | 0.02 | .154 |
| Financial stress | 0.89 | 0.59 | 1.34 | .577 | -0.07 | -0.11 | -0.02 | .008 | -0.10 | -0.16 | -0.04 | .001 | -0.05 | -0.14 | 0.03 | .224 |
| Gender | 0.92 | 0.82 | 1.05 | .219 | 0.03 | -0.03 | 0.09 | .350 | -0.01 | -0.03 | 0.02 | .554 | -0.01 | -0.04 | 0.02 | .400 |
| Age (1=>37years; 0=<38years) | 1.67 | 1.27 | 2.19 | <.001 | 0.06 | 0.01 | 0.11 | .019 | 0.06 | 0.01 | 0.11 | .018 | 0.12 | 0.07 | 0.16 | <.001 |
| Has had prior admissions (1=yes, 0=no) | 1.36 | 0.88 | 2.10 | .169 | -0.09 | -0.15 | -0.03 | .005 | -0.04 | -0.11 | 0.03 | .307 | 0.03 | -0.07 | 0.12 | .573 |
| Number of prior admissions | 0.81 | 0.68 | 0.97 | .021 | -0.01 | -0.10 | 0.07 | .741 | -0.05 | -0.15 | 0.04 | .257 | -0.10 | -0.20 | 0.01 | .073 |
| Race/Ethnicity (ref: Non-Hispanic white) |  |  |  |  |  |  |  |  |  |  |  |  |  |  |  |  |
| Black or African American | 0.38 | 0.24 | 0.59 | <.001 | -0.12 | -0.22 | -0.01 | .032 | -0.08 | -0.13 | -0.03 | .001 | -0.09 | -0.12 | -0.05 | <.001 |
| Hispanic/Latino | 0.76 | 0.48 | 1.20 | .239 | -0.03 | -0.06 | 0.01 | .146 | -0.04 | -0.08 | 0.01 | .108 | -0.02 | -0.05 | 0.01 | .094 |
| Native American/Pacific Islander | 0.85 | 0.63 | 1.15 | .289 | -0.04 | -0.10 | 0.03 | .267 | -0.06 | -0.12 | 0.01 | .092 | -0.05 | -0.12 | 0.02 | .157 |
| Unknown | 0.95 | 0.62 | 1.44 | .808 | 0.00 | -0.04 | 0.03 | .812 | -0.03 | -0.07 | 0.01 | .202 | -0.01 | -0.09 | 0.07 | .799 |
| Buprenorphine as MOUD (ref: Methadone) | 0.36 | 0.19 | 0.66 | .001 | 0.00 | -0.08 | 0.08 | .944 | 0.04 | -0.09 | 0.16 | .557 | -0.13 | -0.22 | -0.04 | .007 |
